# Loss of function in the *Drosophila* clock gene *period* results in altered intermediary lipid metabolism and increased susceptibility to starvation

**DOI:** 10.1007/s00018-019-03441-6

**Published:** 2020-01-20

**Authors:** Stefan Schäbler, Kelechi M. Amatobi, Melanie Horn, Dirk Rieger, Charlotte Helfrich-Förster, Martin J. Mueller, Christian Wegener, Agnes Fekete

**Affiliations:** 1 Pharmaceutical Biology, Julius-Von-Sachs-Institute, Biocenter, University of Würzburg, Julius-von-Sachs Platz 2, 97084 Würzburg, Germany; 2 Neurobiology and Genetics, Würzburg Insect Research, Theodor-Boveri-Institute, Biocenter, University of Würzburg, Am Hubland, 97074 Würzburg, Germany

**Keywords:** Circadian rhythms, Metabolomics, Mitochondrial activity, Tryptophan, Acylcarnitine, Feeding

## Abstract

The fruit fly *Drosophila* is a prime model in circadian research, but still little is known about its circadian regulation of metabolism. Daily rhythmicity in levels of several metabolites has been found, but knowledge about hydrophobic metabolites is limited. We here compared metabolite levels including lipids between *period*^*01*^ (*per*^*01*^) clock mutants and Canton-S wildtype (WT_CS_) flies in an isogenic and non-isogenic background using LC–MS. In the non-isogenic background, metabolites with differing levels comprised essential amino acids, kynurenines, pterinates, glycero(phospho)lipids, and fatty acid esters. Notably, detectable diacylglycerols (DAG) and acylcarnitines (AC), involved in lipid metabolism, showed lower levels in *per*^*01*^ mutants. Most of these differences disappeared in the isogenic background, yet the level differences for AC as well as DAG were consistent for fly bodies. AC levels were dependent on the time of day in WT_CS_ in phase with food consumption under LD conditions, while DAGs showed weak daily oscillations. Two short-chain ACs continued to cycle even in constant darkness. *per*^*01*^ mutants in LD showed no or very weak diel AC oscillations out of phase with feeding activity. The low levels of DAGs and ACs in *per*^*01*^ did not correlate with lower total food consumption, body mass or weight. Clock mutant flies showed higher sensitivity to starvation independent of their background-dependent activity level. Our results suggest that neither feeding, energy storage nor mobilisation is significantly affected in *per*^*01*^ mutants, but point towards impaired mitochondrial activity, supported by upregulation of the mitochondrial stress marker 4EBP in the clock mutants.

**Electronic supplementary material:**

The online version of this article (10.1007/s00018-019-03441-6) contains supplementary material, which is available to authorized users.

## Introduction

The interaction between circadian clocks and metabolism is of increasing interest, since clock dysfunction often correlates with metabolic pathologies [1] and frequent eating causes chronic disruption of the circadian clock and decreases lifespan [2]. For example, animals with disturbed circadian clocks are under high risk to develop diabetes, obesity and other metabolic syndromes [3, 4], and ad libitum access to a high-fat diet causes disruption of the circadian clock and dampens molecular circadian rhythms in rodents [5]. In humans, several metabolites show daily and circadian oscillations [6–8], suggesting that their production or break down is controlled by the circadian clock and that mistiming in those processes may contribute to the above-mentioned health problems. We are, however, far from a complete understanding of which metabolites precisely fall under circadian clock control, and how they are linked to clock-related metabolic disorders. Even more, elucidation of metabolite rhythmicity in relation to the interplay between the central clock in the brain and peripheral clocks in the body is still at an initial stage.

The fruit fly, *Drosophila melanogaster,* provides a genetically tractable model to address the complex interplay between circadian clocks and metabolism [9]. The circadian clock of *Drosophila* is well characterised, and—like in mammals—is closely connected to metabolism. Several metabolites have been shown to oscillate in wildtype fruit flies, but not in *period*^*01*^ (*per*^*01*^) or *timeless*^*01*^ (*tim*^*01*^) mutants. For example, levels of trehalose and glycogen oscillated in wildtype flies under light–dark (LD) and constant dark (DD) conditions, and showed dampened oscillations in the clock mutant *tim*^*01*^, suggesting a link between circadian clocks and carbohydrate storage [10]. Similarly, glutathione levels oscillated in a daily manner in heads of wildtype flies but not in *per*^*01*^ mutants [11]. Consistent with the glutathione fluctuations, levels of mitochondrial H_2_O_2_ also showed daily rhythmicity in heads of wildtype flies but not in *per*^*01*^ mutants under LD [12]. A more detailed NMR-based analysis of cycling metabolites found 14 metabolites (sterols, fatty acids, leucine, valine, isoleucine, alanine, glycine, histidine, tryptophan, creatine, glucose, AMP, NAD, and lactic acid) that oscillated in a daily fashion in wildtype flies subjected to light and temperature cycles [13]. Furthermore, a recent liquid chromatography–mass spectrometry (LC–MS) study on fruit fly metabolome in bodies (thorax and abdomen) of *w*^*1118*^ wildtype-like and *per*^*01*^ mutant flies showed oscillation in profiled polar metabolites [14]. Out of the 236 identified metabolites, levels of 34 oscillated in a daily manner in LD, of which fructose, 3-hydroxybutarate, acetyl-amino sugar, an unknown monosaccharide, gluconate, ribitol/xylol, and riboflavin maintained similar rhythmicity in DD. Remarkably, oscillations of some acylcarnitines (AC) were obviously phase shifted and more robust under DD than under LD [14].

In addition to altered metabolite rhythms, also differences in metabolite levels were reported between wildtype flies and *per*^*01*^ mutants. Disruption of the main circadian pacemaker neurons in the fly brain leads to increased levels of triacylglycerols (TAGs) in the fat body, the counterpart of the mammalian liver and adipose tissue [15]. TAG levels in the body of arrhythmic *tim*^*01*^ mutants were reduced under caloric restriction compared to wildtype female flies. In addition, low-caloric food increased the amplitude and robustness of clock gene oscillations in the fat body [16].

Notwithstanding this recent progress, the circadian regulation of lipid metabolism in *Drosophila* remains largely unexplored. This is in particular true for the intermediate diacylglycerols (DAGs, the mobilized form of TAGs in insects), and ACs which are the transport forms of fatty acids into the mitochondria. ACs were already shown to display circadian oscillations in w^1118^ wildtype-like flies [14], yet, we lack a detailed description on the valence and interaction of circadian clocks and different AC species at high molecular resolution. The same applies for other catabolic pathways used for energy gain, including sugars and amino acids. Moreover, it seems that the comprehensive studies looking at lipids and ACs [14, 16] all compared flies with a non-isogenic background. As the genetic background may affect overall metabolic features, the extent to which observed differences are due to general background effects remains elusive.

To help filling these gaps in knowledge and to meet the need for a higher number of chronometabolomic studies [17], we compared metabolite profiles including lipids between *per*^*01*^ mutant and wildtype flies (wildtype Canton S, WT_CS_) in a non-isogenic and isogenic background using LC–MS-based metabolomics. Identified metabolites differing between the two genotypes were further investigated if their levels displayed time of the day-dependent changes in wildtype flies and additionally, they were correlated to resistance to starvation and to the related circadian-controlled behaviours feeding and locomotor activity. Our results indicate that a null mutation in *per* does not broadly affects carbohydrate and lipid metabolism, but tryptophan and specific lipids. These substances were also identified as clock-dependent in mice [17] and hence may represent basal core clock-dependent metabolites.

In combination with increased starvation susceptibility and increased stress marker level in *per*^*01*^ mutants, our data point towards altered mitochondrial activity in flies with a perturbed clock.

## Material and methods

### Fly rearing and sampling

We used Canton S (WT_CS_) as wildtype control, and *w*^+^ *per*^*01*^ [18, 19] as clock mutation which is widely characterized at the behavioral/physiological level and leads to a disruption of the molecular clock [20]. Both strains were a kind gift of Bambos Kyriacou (Leicester) and possess different backgrounds. We initiated the analysis with these non-isogenic flies. To account for genetic background effects, and since chronometabolomic studies are prone to low reproducibility due to physiological and technical variability [17], we additionally used highly isogenic WT_CS_ and *per*^*01*^ flies that were mutually backcrossed for 37 (*per*^*01*^) and 54 (WT_CS_) generations [21] for control experiments regarding metabolites that significantly differed between the non-isogenic genotypes. In the following, the non-isogenic and isogenic flies are referred to as _n_*per*^*01*^/_n_WT_CS_ and _i_*per*^*01*^/_i_WT_CS_, respectively. *per*^*01*^/WT_CS_ is used when both genetic backgrounds are referred to.

Male flies were reared under 12/12 h LD at 25 ± 0.2 °C and 60 ± 2% relative humidity. Standard food consisted of 0.8% agar, 2.2% sugar beet syrup, 8.0% malt extract, 1.8% yeast, 1.0% soy flour, 8.0% corn flour, and 0.3% hydroxybenzoic acid. 5-day-old flies were collected at different Zeitgeber times (ZT) and were shock frozen in liquid nitrogen (− 80 °C) immediately after sampling. To immediately lock the metabolic state, gut content was not emptied before sampling. This handling might have resulted in higher levels of PCs and PEs and a higher PC/PE ratio compared to studies using starved animals [22]. Fly heads and bodies were separated on ice and five heads or bodies were pooled per sample and stored at − 80 °C until extraction.

### Analysis of hydrophilic and lipophilic metabolites

Fly heads and bodies were extracted using liquid–liquid extraction to recover polar and apolar metabolites from the same sample using an adapted protocol [23]. The extraction solvent, methanol containing internal standards, was added to the sample (200 µl for heads and 400 µl for bodies) which was homogenised with a bead-based tissue homogeniser for 5 min at 23 Hz. After transferring samples to an ultrasonic bath for 10 min, 1 ml of methyl *tert*-butyl ether and 250 µl of water were added and the homogenate was incubated for 1 h at room temperature on a rotating shaker. To obtain clear separation of the two phases, samples were kept for 10 min at room temperature and then centrifuged at 11,363 g for 10 min. Organic phases and aqueous phases were transferred separately into reaction tubes and dried until complete dryness under vacuum. Prior to LC–MS analysis, the dried organic phases were reconstituted in isopropanol (100 µl), while 80% methanol containing 0.1% formic acid (75 µl into head samples, 150 µl into body samples) was added to the dried aqueous phases.

LC–MS analysis was performed using an Acquity Ultra Performance LC coupled to a Synapt G2 HDMS equipped with electrospray ionisation source (Waters). Lipophilic metabolites in the organic phases were separated by reversed phase LC, while hydrophilic metabolites in the aqueous phases were analysed by hydrophilic interaction LC. LC separation of the organic phase was used for untargeted lipidomics, profiling of TAGs, DAGs, and structural lipids according to an adapted protocol [24]. Analysis of the aqueous phase was used for metabolite fingerprinting of polar metabolites and for quantification of amino acids according to an adapted protocol [25]. MS was operated in positive electrospray ionisation mode and centroid data were acquired with a mass range from 50 to 1200 Da using leucine–enkephaline for internal mass calibration. MassLynx 4.1 was used to operate the instrument and operational parameters are listed in Suppl. Table 1. Head and body samples were measured independently in different batches. All samples were measured in a randomized fashion, and quality control samples (pooled sample from all head/body samples) were measured every seventh measurement to assess instrument performance.

After measurements, raw data were transferred to the NetCDF format by DataBridge (Waters) and preprocessed using the R-based XCMS package [26]. Centwave algorithm [27] was used for peak detection and default values were used except for ppm (15), peakwidth_min (6), peakwidth_max (aqueous phase: 60 and organic phase: 15) and snthresh (aqueous phase: 10 and organic phase: 25). For grouping of detected features across samples bw was set to 5, minfrac to 0.75, and mzwid to 0.03. Retention time correction was performed using the obiwarp method with the default parameters [28]. For lipid profiling, internal standards were applied to determine levels of the identified lipid species. The response factor was set to one, since authentic reference material for all identified glycerolipids and phospholipids was not available.

### Statistical analysis and feature identification

In the untargeted experiment, differing metabolites were identified using the wrapper function annotate Diffreport, which uses the diffreport function of XCMS and combines it with the feature annotation of the R package CAMERA [29]. The detection of differing peaks was performed in a body part- and time point-specific manner using univariate statistical analysis (Welch t test). To ensure that detected differences in metabolite levels are due to the mutation in *per* and not due to experimental factors, the whole experiment was performed twice, and only those features were considered as differing metabolites when they were detectable in both experiments and differed significantly. Tolerance values between the two experiments with mass difference of 10 mDa, retention time shift of 60 s for aqueous phase analysis and 10 s for organic phase analysis were permitted.

In the time course experiment, feature levels had to be normalized since quality control sample measurement revealed ion suppression throughout the analysis of apolar metabolites. Therefore, we used a method [30] where intensities were corrected feature wise such that the mean intensity of the samples was the same for each batch, with one replicate of all time points collected over 3 days representing one batch. JTK_CYCLE algorithm was used to identify oscillations at period length of 24 h in metabolite levels [31]. Metabolites were considered to show a daily oscillation if both the adjusted p value (adj.p) and the Benjamini–Hochberg false discovery rate (BH.Q) were below 0.05. Next to BH.Q and adj.p, JTK_CYCLE also predicts the characteristics of an oscillation-like amplitude (AMP) and maximum, termed as lag phase (LAG).

To aid identification of metabolites, grouping of features according to their retention time and peak shape, obtained by CAMERA [29], was used. The annotation of the CAMERA package was manually inspected and elemental compositions of molecular ions were calculated using an elemental composition calculator, implemented in MassLynx v.4.1 (Waters). To elucidate the structure, mass-to-charge ratios of molecular ions generated in low energy function and fragmentation patterns generated in high energy function were compared with METLIN Metabolomics Database [32]. To unequivocally identify the metabolites and achieve a level 1 identification according to the Metabolomic Standard Initiative [33], retention times, ionisation behaviours, and fragmentation patterns of the measured peak and reference material were compared at identical analytical conditions.

### LC MS/MS profiling of carnitines

For the analysis of carnitines (carnitine and ACs), methanolic extraction was used since most ACs were detectable in both phases when liquid–liquid extraction was applied. Extraction solvent was cold methanol containing 10 mM ammonium acetate, 0.3% formic acid, and internal standards. Extraction volume was 200 µl for head samples and 400 µl for body samples. Homogenization was performed with a bead-based tissue homogeniser (23 Hz, 5 min) before samples were transferred to an ultrasonic bath for 10 min. Afterwards, samples were incubated for 10 min on ice, then centrifuged at 11,363 g for 10 min to remove precipitated methanol-insoluble proteins and inorganic salts. Finally, 50 µl of the supernatant were dried until completeness under vacuum, and were resuspended in 95% acetonitrile containing 10 mM ammonium acetate and 0.3% formic acid. ACs were analysed using an Acquity Ultra performance LC (Waters, Milford, USA) coupled to a Sciex 6500 + QQQ-MS (AB Sciex) using an adapted protocol [34]. Analyst 1.6.3 was used to operate the instrument and to integrate peaks identified as carnitine or ACs. Levels of identified AC species were determined using internal standards and a response factor of one. Analysis parameters are listed in Supplementary Table 1.

### Analysis of sugars, glycogen and proteins

For the analysis of sugars, the same extraction protocol was performed as for ACs except that the extraction solvent was 50% acetonitrile. Operational parameters of the LC–MS analysis are listed in Supplementary Table 1. Our LC–MS analysis revealed 3–4 times higher levels of glucose than trehalose, which differs from previous reports [35]. These differences are likely due to the different applied extraction methods and glucose contents of the food. For the analysis of glycogen, remaining extracts and water extracts of pellets (100 µl, ball mill: 23 Hz for 5 min, sonic bath for 15 min) were pooled together. Glycogen levels were determined according to an adapted protocol [35] with the exception that we used Amplex Red Kit (Invitrogen) for fluorescent determination of glucose. Bradford assay was applied for protein quantification [36]. Prior analysis, head and, body samples were extracted first in 400 µl water and then in 400 µl 0.1 M sodium hydroxide on a ball mill at 23 Hz for 5 min, followed by ultrasonication for 10 min.

### Feeding assay, locomotor activity, and starvation assay

To determine food consumption, a modified CAFE assay was used [37]. Small cylindrical vials (4.5 × 1.4 cm) served as chambers and were filled with 1 ml of 2% agar which provided water and constant humidity. Five holes were made on the plastic cover with one at the center. The center hole hosted a 30-µl food capillary tube. One of the side holes hosted a 30-µl control capillary tube which was inaccessible to the flies and used to measure the evaporation rate. Each capillary had a 1-µl oil overlay to reduce evaporation. The food capillary was filled with 3.6% (w/w) yeast, 5% (w/w) sucrose containing 0.3% (v/v) FD&C Blue No 1 (E133) blue as coloring agent and food was changed every 2 days between ZT20 and ZT22. Ten flies were placed within each CAFE chamber, and pictures were taken every hour for at least eight consecutive days after eclosion by a DMK22BUC03 video camera with a Pentax C2514-M objective in combination with IC capture software (www.theimagingsource.com). From the pictures, we then measured the distance L_d_ between the food menisci from hour to hour, corrected for evaporation and calculated the volume of consumed food as L_d_* capillary volume/total length of the capillary.

For recording locomotor activity, commercial *Drosophila* Activity monitors (Trikinetics) were used as described previously [38]. Individual male flies were first recorded for 7 days under LD cycles (with 100 lux intensity during the day) and subsequently for 14 days under DD. Temperature and humidity were kept constant during the experiment (25 °C ± 0.2 °C; 60% ± 2% rH). Average activity profiles (beam crosses/minute) were calculated for *per*^*01*^ and WT_CS_ under LD (day 2 and 6) and for day 1 and 2 in DD. Under LD cycles, the daily average activity levels were calculated for both strains. Data were analysed by a Shapiro–Wilk normality test, followed by a Kruskal–Wallis and pairwise Wilcoxon signed-rank test using the R commander package (Rcommander.com).

For the starvation experiment, locomotor activity tubes were filled with 3% sugar free agar. 1- to 3- day-old male WT_CS_ and *per*^*01*^ flies were transferred from the standard food to the starvation tubes at two different time points: between ZT0 and ZT1 (morning) and between ZT6 and ZT7 (afternoon). Consequently, the first group of flies had no access to food after ZT0 and the second after ZT6. Fly activity was then registered in the activity monitors as described above (under LD at 25 °C ± 0.2 °C; 60% ± 2% rH). As starving flies are continuously moving [39, 40], we could reliable judge the exact time of their death by measuring the time (in h) that passed until they stopped moving. The survival function was analysed using a Kaplan–Meier estimator, followed by a Breslow test implemented in the software package OriginPro 2019 (OriginLab, Northampton MA, USA).

### Quantitative real-time RT-PCR (qPCR)

For quantification of metabolic gene mRNA expression, total mRNA was extracted from ten heads or five bodies (thorax plus abdomen) of _n_WT_CS_ and _*n*_*per*^*01*^ flies at ZT06 and ZT18 using the Quick-RNA MicroPrep Kit (Zymo Research, for heads) and the Quick-RNA Tissue/Insect RNA MicroPrep Kit (Zymo Research, for bodies) according to manufacturer’s instructions. Tissues were collected in 300 (heads)/800 (bodies) µl RNA lysis buffer. Heads were homogenised with a plastic pestle; bodies were homogenised using beads. Total RNA was eluted in 8-µl RNAse-free water. For cDNA synthesis, 1-µg mRNA and the QuantiTect Reverse Transcription Kit from Qiagen were used according the manufacturer’s protocol. Genomic DNA was removed by adding 1 µl of gDNA wipeout buffer to 6 µl of the eluted RNA. After incubation at 42 °C for 2 min, samples were placed for 2 min at 4 °C and 6 μl of a mastermix composed of 4 μl RT Buffer, 1 μl RT Primer Mix, and 1 μl reverse transcriptase was added. Reverse transcription was performed for 30 min at 42 °C, followed by 3 min at 95 °C and 2 min at 4 °C. Finally, 80 μl of water was added and cDNA samples were stored at − 20 °C. For qPCR, 2-µl cDNA per probe was amplified on a Rotorgene Q cycler (Qiagen), using the Bioline SensiFAST SYBR No-ROX kit and an annealing temperature of 60 °C. RpL23 served to normalise transcription levels. Data were analysed by Q-Rex software (Qiagen). The following primer pairs were used: *4EBP* fw: CCAGGAAGGTTGTCATCTCG, rv: CCAGGAGTGGTGGAGTAGAGG; *bmm* fw: GGTCCCTTCAGTCCCTCCTT, rv: GCTTGTGAGCATCGTCTGGT; *RpL23* fw: GACAACACCGGAGCCAAGAACC, rv: GTTTGCGCTGCCGAATAACCAC; *sxe2* fw: TGCGGTACGATCTTTATACGCC, rv: CTAACTGGCCATTTCGGATTGA; *HNF4* fw: CTGTCCAGATCCCCTTGTGT; rv: GGCAGGATGAGCAGAATCTC. Relative expression was calculated as 2^−ΔΔCT^ [41] and normalised to the respective value of _n_WT_CS_ at ZT6. Results were analysed by a Shapiro–Wilk normality test, followed by a Kruskal–Wallis and pairwise Wilcoxon signed-rank test using the R commander package (Rcommander.com).

## Results

### A mutation in the clock gene *period* affects intermediates of storage lipid breakdown

To investigate whether the circadian clock affects lipid metabolism, we first compared the lipid profile between fed clock mutant and wildtype flies. We sampled heads and bodies of male _n_*per*^*01*^ and _n_WT_CS_ flies at ZT6 and ZT18, since levels of *period* mRNA are at a trough in the middle of the light phase and peaks in the middle of the dark phase in wildtype flies kept under LD [42]. Heads and bodies of male _i_*per*^*01*^ and _i_WT_CS_ flies were sampled at ZT6. After extraction, main structural lipids [diacylglycerophosphoethanolamines (PEs), diacylglycerophosphocholins (PCs)], TAGs, DAGs, and ACs from the organic phase were determined by LC–MS. Univariate statistical analysis revealed significantly different levels in 130 out of 141 detected lipids between the two non-isogenised genotypes in at least one body part and at one sampling time point. Total levels of differing PEs and PCs were low and differences between the genotypes were small (Suppl. Figure 1). This suggests that levels of structural lipids are not drastically altered by disturbing whole body clock function, at least not in young male flies.

**Fig. 1:**
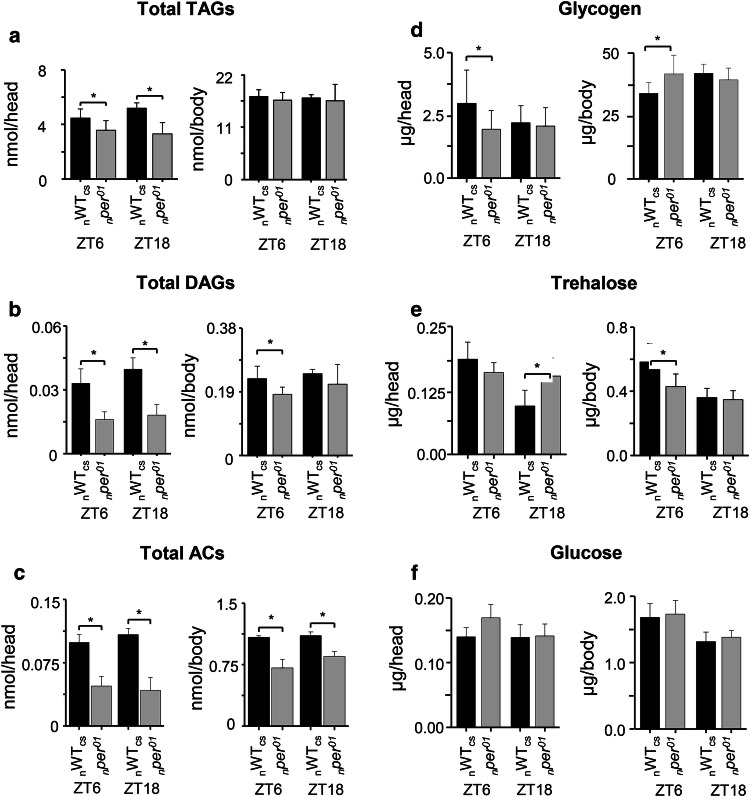
Comparison of metabolite levels in heads and bodies between _n_WT_CS_ (black bars) and _n_*per*^*01*^ flies (gray bars) at ZT6 and ZT18. Differences were observed between **a** total TAG level summed over all TAG species, **b** total DAG level summed over all DAG species, **c** total AC level summed over all AC species. **d** Glycogen, **e** trehalose, and **f** glucose showed wildtype-like levels in the clock mutant. Asterisks denote statistically significant differences (*p* < 0.05, *t* test) between the genotypes. Data represent means ± s.d., *n* = 7

The total level of TAGs differed only between heads, but not bodies of *per*^*01*^ and WT_CS_ flies (Fig. 1a). While total TAG levels thus were not consistently affected in *per*^*01*^ mutants, a tendency to lipid remodeling in the TAG pool in the head was present. Levels of TAGs with fatty acyl carbon between 34 and 49 were lowered, whereas several TAGs with unsaturated fatty acyl carbon higher than 50 were higher in heads of *per*^*01*^ flies compared to WT_CS_ in both genetic backgrounds (Suppl. Figure 2).

In contrast to TAGs, significantly lower levels of DAGs were found in the body of *per*^*01*^ mutants compared to WT_CS_ at ZT6, irrespective of the genetic background (Fig. 1b). Furthermore, the total level of ACs, which are intermediates of lipid metabolic pathways, was significantly decreased in both heads and bodies of _*n*_*per*^*01*^ mutants relative to _n_WT_CS_ at both ZT6 and ZT18 (maximum fold difference 0.3, Fig. 1c). This decrease was due to reduced levels across the carnitine and ACs species in _*n*_*per*^*01*^ mutants, except for propionyl–carnitine [AC(C3:0)] which was higher in the _*n*_*per*^*01*^ mutant (Fig. 3a). A significantly reduced total AC level could not be confirmed for _*i*_*per*^*01*^ flies at ZT6 due to carnitine and short-chain AC levels which did not differ compared to _i_WT_CS_ flies (Suppl. Figure 3). A closer look on the different AC species in _*i*_*per*^*01*^*, *however, revealed again a strong and significant reduction across long-chain ACs as in the _*n*_*per*^*01*^ mutant (Fig. 3b). In addition, the significant increase of propionyl–carnitine in the *per*^*01*^ mutant persisted in the isogenic background (Fig. 3b). Taken together, these results suggest that the *per*^*01*^ mutation negatively and broadly affects long-chain AC levels in the body, and leads to increased levels of propionyl–carnitine while other short-chain ACs were not affected.

**Fig. 2:**
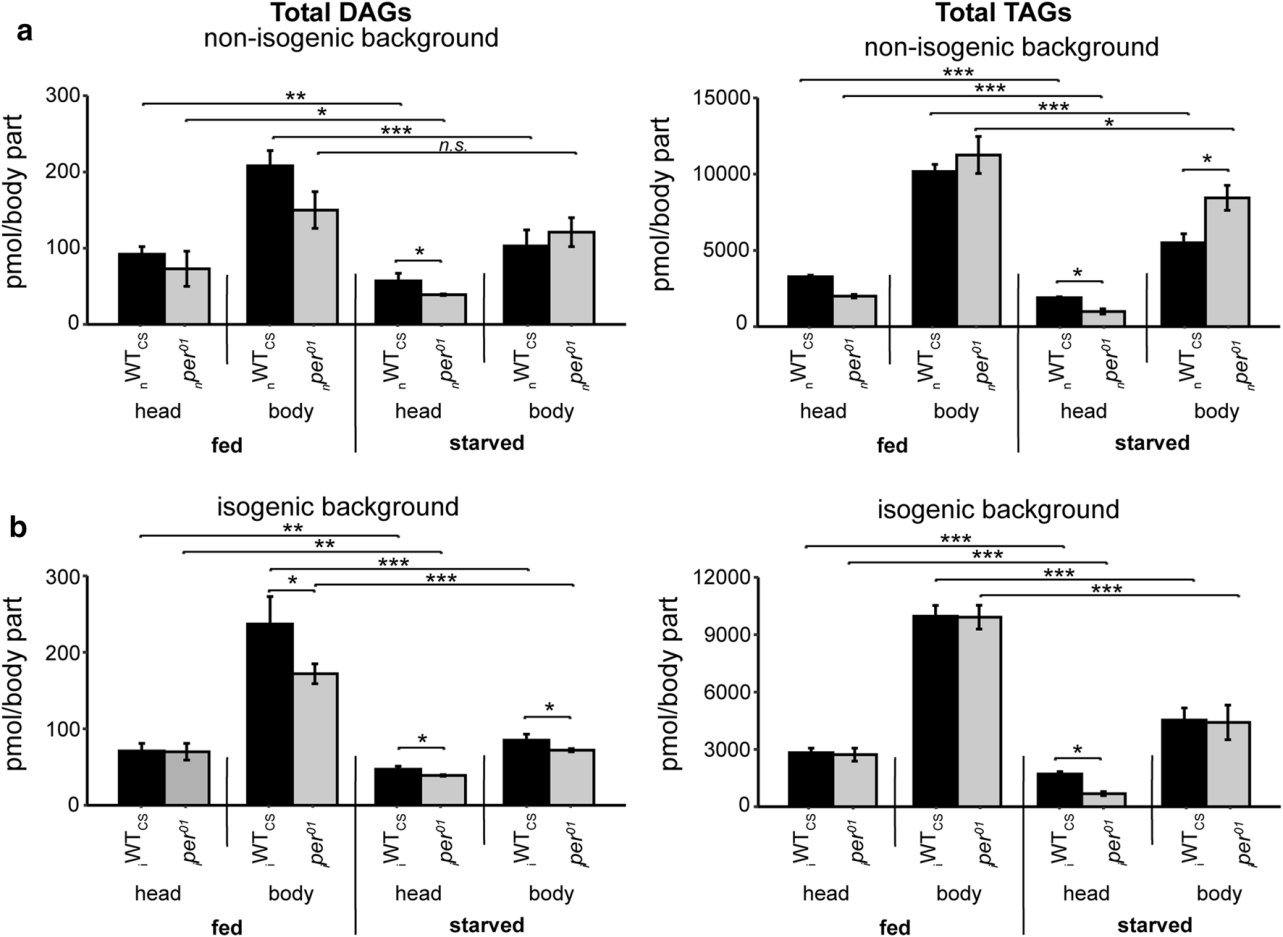
DAG (left) and TAG (right) levels between WT_CS_ (black bars) and *per*^*01*^ (gray bars) flies in the **a** non-isogenic, and **b** isogenic background and feeding state at ZT6. Asterisks denote statistically significant differences (*p < 0.05, **p < 0.01, ***p < 0.001,* t* test) between the genotypes. Data represent means ± s.d.,* n* = 4

To test whether lowered DAG and AC levels in *per*^*01*^ flies might be linked to deficits in lipid mobilisation, we starved flies for 24 h and then compared total TAG levels with those obtained in fed flies (Fig. 2). In both genetic backgrounds, *per*^*01*^ as well as WT_CS_ flies showed reduced TAG levels upon starvation. There was no significant difference in the extent of TAG level reduction upon starvation between *per*^*01*^ mutants and WT_CS_ controls. These results suggest that lipid mobilisation upon starvation is not significantly affected by an impaired clock.

For lipid profiling, we had to define metabolites of interest beforehand. Lipids other than the identified structural lipids, TAGs, DAGs, and ACs may thus have escaped detection. Therefore, we additionally performed metabolite fingerprinting where relative intensities of all detectable peaks of two independently performed experiments were compared. Based on retention times and mass-to-charge ratios, we found 140 peak-aligned metabolite features in heads and bodies which significantly differed (*p* < 0.05) between _n_*per*^*01*^ and _n_WT_CS_ flies (Suppl. Table 2). Out of these, structures of 60 lipids could be elucidated employing database search (Suppl. Table 3). The majority of the differing lipids were the already profiled lipids (PEs, PCs) and metabolites of storage lipid breakdown (DAGs, ACs). Taken together, our results from lipid profiling and metabolite fingerprinting of the organic phase of fly extracts suggest that a nonfunctional *per* gene has an impact on processes involving glycerolipids and ACs, while structural lipids are only little affected.

### *per*^*01*^ affects the level of tryptophan, but not other amino acids and related metabolites

**Fig. 3:**
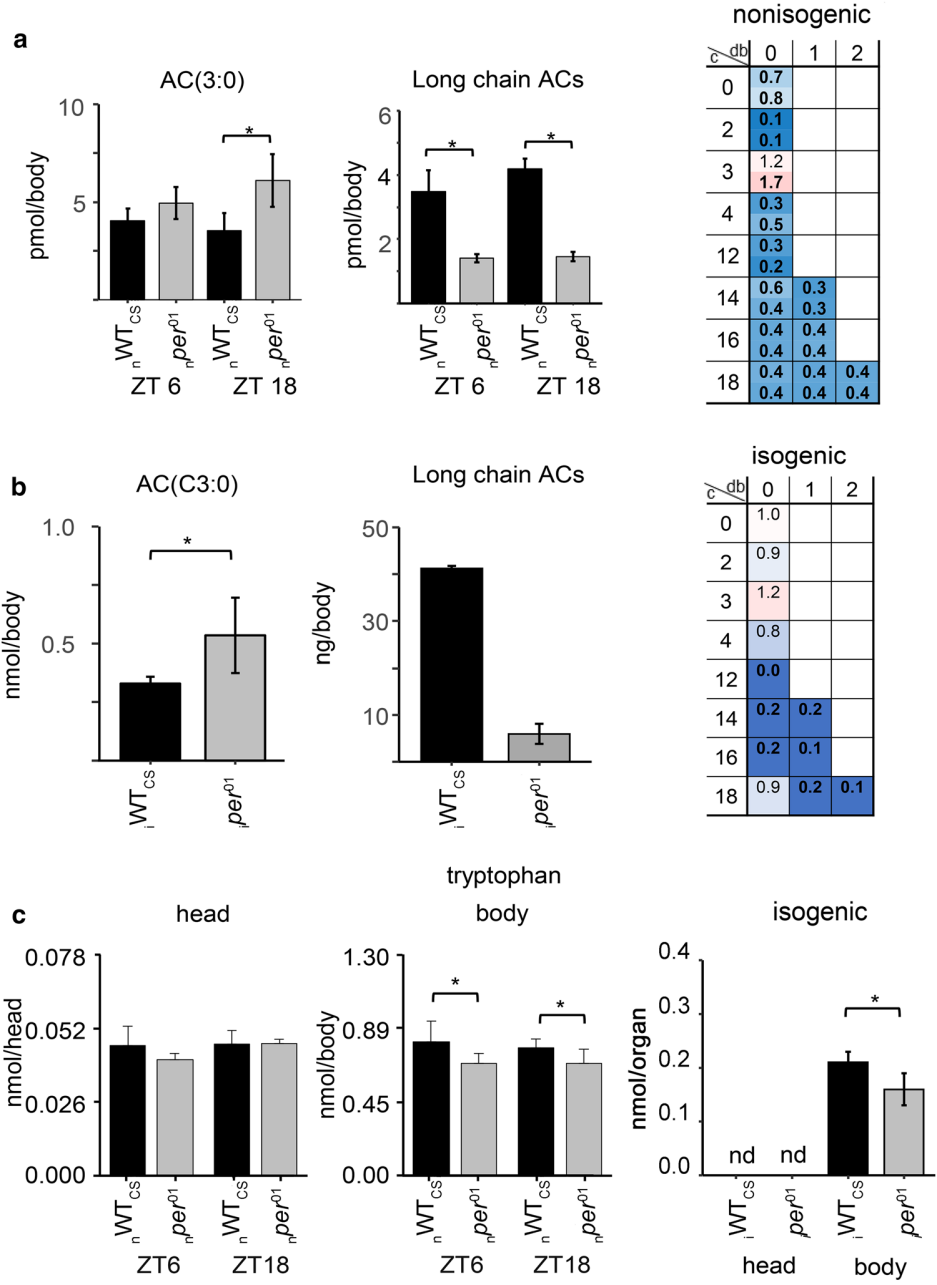
Comparison of **a–b** AC and **c** tryptophan levels between WT_CS_ (black bars) and *per*^*01*^ (gray bars) flies. **a** AC3:0 and total long-chain AC levels in the non-isogenic background at ZT6 and ZT18. **b** AC3:0 and total long-chain AC levels in the isogenic background at ZT6. **c** Tryptophan levels in the head (left) and body (middle) of non-isogenic flies, and in isogenic flies (right). Asterisks denote statistically significant differences (*p* < 0.05,* t* test) between the genotypes. Data represent means ± s.d.,* n* = 4

Since lipid profiling revealed different levels of intermediates in lipid metabolic pathways (DAG, AC) in *per*^*01*^ compared to WT_CS_ flies, we tested whether other metabolites were likewise affected by a nonfunctional *per* gene. First, we determined the storage forms of carbohydrates (glycogen) in heads and bodies of _*n*_*per*^*01*^ and _n_WT_CS_ flies. As in the case of TAGs, glycogen (Fig. 1d) and total protein content (_n_WT_CS_: 74 ± 7 µg/fly, _*n*_*per*^*01*^: 80 ± 8 µg/fly at ZT6) were wildtype-like in _*n*_*per*^*01*^. Next, we compared the products of glycogen breakdown (trehalose, glucose) and protein breakdown (20 canonical amino acids) between heads and bodies of _n_*per*^*01*^ and _n_WT_CS_ flies using targeted metabolomics. The levels of glucose and trehalose were wildtype-like in _*n*_*per*^*01*^ flies except for trehalose in heads at ZT18 (Fig. 1e,f). Though not shown, pilot experiments revealed a considerable variability in trehalose levels and do not confirm a consistent difference in heads or bodies at the two time points. We, therefore, conclude that carbohydrate levels are not significantly affected in _*n*_*per*^*01*^ flies. Also the levels of non-essential amino acids are largely unchanged (Suppl. Figure 4). The levels of six (valine, leucine/isoleucine, phenylalanine, histidine, methionine, and tryptophan) out of eight essential amino acids were lower in _*n*_*per*^*01*^ flies compared to _n_WT_CS_ (Fig. 3c, Suppl. Figure 5). Methylthioadenosine, a product of the polyamine metabolism that affects various metabolic and cellular processes including cell growth [43, 44], was also lower in bodies of _n_*per*^*01*^ relative to _n_WT_CS_ (Suppl. Figure 6e). The differences for essential amino acids are unlikely to be related to the *per* mutation as differences observed in non-isogenic flies were absent between _*i*_*per*^*01*^ and _i_WT_CS_ flies and vice versa (Suppl. Figure 7), with exception of the significant reduction in body tryptophan levels which persisted also in the isogenic background (Fig. 3c).

We, therefore, performed metabolite fingerprinting for the aqueous phase of head and body extracts (Suppl. Table 2), and found significantly lower levels of metabolites in the kynurenine pathway of tryptophan metabolism (kynurenine, hydroxyl kynurenine, kynurenic acid, and xanthurenic acid. Suppl. Figure 6a–d) in bodies of _n_*per*^*01*^ compared to _n_WT_CS_. This is in line with the observed decreased levels of tryptophan in bodies of _n_*per*^*01*^ flies found by the targeted approach (Fig. 3c). In addition, significantly differing levels were determined for pteridines (pterin, sepapterin, and biopterin) between the two non-isogenic genotypes (Suppl. Figure 6f–h). Biopterin was one of the rare differing metabolites for which the level was higher in _*n*_*per*^*01*^ relative to _n_WT_CS_ flies (fold difference of 1.2). Pterins participate in biological oxidations, with tetrahydrobiopterin acting as a cofactor for aromatic amino acid monooxygenases including tryptophan monooxygenase, a key enzyme for tryptophan degradation. _n_*per*^*01*^ flies thus seem to possess an altered tryptophan metabolism. Surprisingly, biopterin levels were significantly higher in _i_WT_CS_ flies compared to _*i*_*per*^*01*^ mutants and pterin and xanthurenic acid levels did not differ (Suppl. Figure 8). Only sepiapterin levels were consistently reduced in the body of *per*^*01*^ mutants in both genetic backgrounds. Our results thus lend little support to an effect of an impaired clock onto tryptophan metabolism, and do not provide a clue as to why tryptophan levels were consistently lower in the *per*^*01*^ mutants.

Confirming the unaltered glucose and trehalose levels in _*n*_*per*^*01*^ mutants using targeted analysis, we did not detect significant differences in metabolites involved in carbohydrate and amino acid metabolism (except tryptophan) between the two non-isogenic genotypes using metabolite fingerprinting approach.

### Acylcarnitines show robust diurnal oscillations in heads and bodies

So far, our results showed that general levels of ACs, DAGs (Figs. 1, 2, 3), and tryptophan (Fig. 3c) were affected by a nonfunctional *per* gene in *Drosophila*. Next, we tested whether the *per*^*01*^ mutation affects possible diel oscillations in these and other metabolites. We reared _n_WT_CS_ flies under LD condition and ad libitum access to standard food, and collected heads and bodies every 2 hours for three consecutive days. Differing metabolites were analysed in raw extracts by LC–MS and daily rhythmicity was analyzed using JTK_CYCLE [28]. Out of the 153 differing metabolites, 56 were considered as rhythmic (Suppl. Table 4), of which 21 oscillated with a relative amplitude (AMP.rel) higher than 10% (Table 1). Among these 21 oscillating metabolites, we identified eleven ACs, the amino acids valine and tryptophan, three DAGs as well as four TAGs. Moreover, levels of further 19 TAGs showed daily rhythmicity, especially in bodies of _n_WT_CS_ (Suppl. Figure 9). This result obtained on nutrient-rich standard food confirms the results of an earlier report for female flies under dietary restriction [16]. In addition to the 23 oscillating TAGs, eight out of eleven DAGs showed diel oscillations in _n_WT_CS_ (Suppl. Figure 9). All oscillating long-chain DAGs and seven oscillating long-chain TAGs with a total acyl carbon ≥ 48 and double bonds ≥ 2 peaked between ZT18 and ZT4, whereas the other 15 oscillating shorter chain TAGs with a total acyl carbon ≤ 47 and double bonds 0–2 peaked at ZT14 in bodies of _n_WT_CS_ (Suppl. Table 4).

**Table 1:** Metabolites which levels differed between *per*^*01*^ and WT_CS_ and showed daily rhythmicity with relative amplitude higher than 10%

	BH.Q	ADJ.P	LAG	AMP.rel
Octadecadienylcarnitine (AC(18:2)) in head	1.32E-45	1.02E-46	2	33
Tetradecanoylcarnitine(C14:0) in head	8.32E-12	5.76E-12	5	28
Tetradecenoylcarnitine(C14:1) in head	8.14E-20	3.76E-20	4	26
Palmitoylcarnitine(C16:0) in head	3.11E-36	4.78E-37	4	26
Palmitoleoylcarnitine(C16:1) in head	2.91E-24	8.96E-25	4	25
Oleoylcarnitine(C18:1) in head	2.22E-26	5.13E-27	4	23
Dodecanoylcarnitine(C12:0) in head	2.66E-11	2.05E-11	4	22
Acetylcarnitine(C2:0) in head	3.55E-14	2.19E-14	4	19
TAG(48:4) in head	1.37E-02	1.35E-03	23	17
Propionylcarnitine(C3:0) in head	2.48E-09	2.10E-09	22	15
Valine in head	2.25E-03	5.63E-04	8	14
Butyrylcarnitine(C4:0) in head	4.77E-22	1.84E-22	22	14
Stearoylcarnitine(C18:0) in head	7.13E-16	3.84E-16	3	13
Tryptophan in head	2.27E-02	1.14E-02	0	11
TAG(50:5) in body	7.03E-05	7.35E-06	18	25
TAG(48:4) in body	1.62E-02	5.36E-03	21	16
DAG(30:2) in body	3.95E-03	4.93E-04	19	15
Valine in body	8.16E-05	6.28E-06	8	15
TAG(52:5) in body	1.62E-06	2.42E-08	22	15
DAG(28:0) in body	2.47E-02	9.28E-03	4	13
Octadecadienylcarnitine(C18:2) in body	1.25E-08	1.92E-09	3	12
DAG(34:1) in body	4.66E-03	8.74E-04	0	12
Oleoylcarnitine(C18:1) in body	5.41E-07	1.67E-07	4	11
TAG(50:1) in body	2.00E-02	7.17E-03	22	11
PE(31:1) in body	8.94E-03	1.34E-03	14	11
Palmitoylcarnitine(C16:0) in body	3.63E-09	2.80E-10	3	10

A metabolite was considered to display daily rhythmicity if BH.Q and adj.p were below 0.05 at a period length of 24 h using JTK_Cycle algorithm. Strong oscillations of ACs in heads and glycerolipids in bodies of WT_CS_ under LD. Oscillation parameters of metabolites differing between *per*^*01*^ and WT_CS_ (period length of 24 h and relative amplitude higher than 10%)

*BH.Q* Benjamini–Hoechberg false discovery rate, *ADJ.P* adjusted p value, LAG: lag phase (maximum), *AMP.rel* relative amplitude [%], *AC* acylcarnitine, (*X:Y*) X is the number of C atoms and Y is the number of double bonds in the esterified fatty acids, *TAG* triacylglycerol, *DAG* diacylglycerol, *PE* diacylglycerophosphoethanolamine

**Fig. 4:**
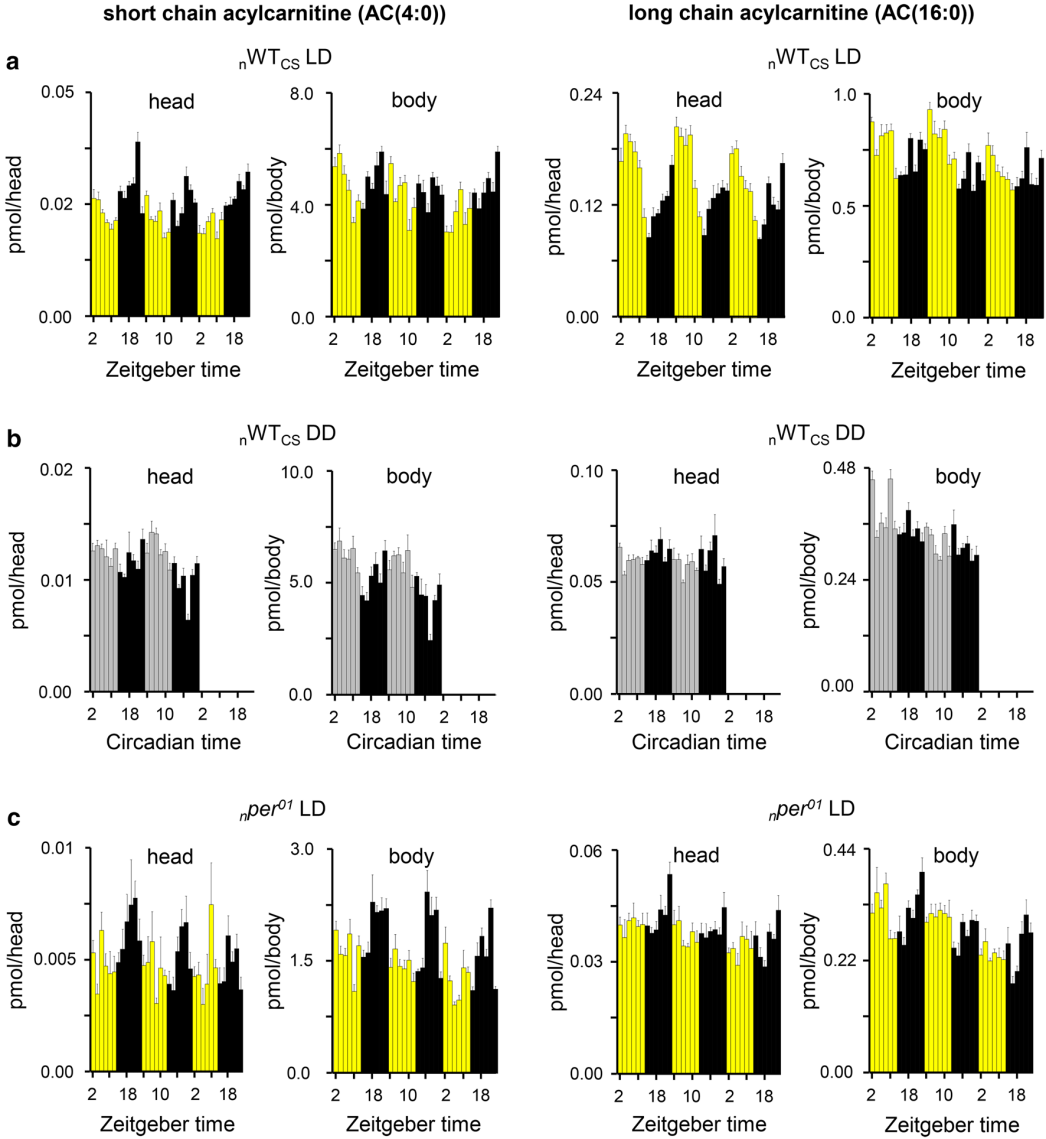
The diel rhythm in AC levels in WT_CS_ under LD virtually dampened under DD and in *per*^*01*^ under LD. Daily variations in AC(4:0) (left) and AC (16:0) (right) levels were determined in **a** WT_CS_ under LD, **b** in WT_CS_ under DD, and **c** in *per*^*01*^ at LD (**c**). Heads (left plots) and bodies (right plots) were collected every 2 hours for three consecutive days. Data represents mean ± s.e.m., *n* = 4–7

ACs showed even more pronounced and robust diurnal oscillations than TAGs and DAGs (Suppl. Table 4) in _n_WT_CS_ in LD conditions. For instance, the lowest BH.Q value was 10^–45^ for AC(18:2) and 10^–6^ for TAG(39:0) (Table 1). In _n_WT_CS_ heads, long-chain ACs (12:0, 14:0, 14:1, 16:0, 16:1, 18:0, 18:1, 18:2), short-chain ACs (2:0, 3:0, 4:0) as well as carnitine strongly oscillated (BH.Q < 10^–7^ and AMP.rel > 10%) with a maximum between ZT22 and ZT5 at the transition between dark and light phase (Suppl. Table 5). Similar oscillation profiles were also found for most ACs (2:0, 4:0, 16:0, 16:1, 18:0, 18:1, 18:2) in bodies of _n_WT_CS_ (Suppl. Figure 10). Representative examples for diel rhythms of short- and long-chain ACs (AC(4:0) and AC(16:0)) are given in Fig. 4.

These results show that AC levels oscillate with robust daily rhythmicity in wildtype flies under LD (Suppl. Table 4). To identify whether these oscillations are endogenous and clock-controlled, or driven by the external light cycle, we next determined AC levels in _n_WT_CS_ under DD and in _*n*_*per*^*01*^ under LD (Fig. 3b, c). In _n_WT_CS_, short-chain ACs (2:0, 3:0, 4:0) continued to cycle in a daily fashion even in DD, though the maxima were phase shifted by ~ 6 h (Suppl. Figure 10, Suppl. Table 5). Rhythms of long-chain ACs dampened in _n_WT_CS_ under DD, with the exception of AC(14:0) and AC(16:1) in _n_WT_CS_ heads which continued to cycle with a shifted phase (Suppl. Figure 10, Suppl. Table 5). In heads of _*n*_*per*^*01*^ mutants under LD, AC levels did neither cycle nor showed strongly weakened oscillations. BH.Q values increased with a magnitude order of 4–39 except for AC(2:0) and AC(18:0) in bodies. We conclude from these finding that the oscillations of most ACs are endogenous, and likely controlled–at least in part—by peripheral clocks which could explain the dampened AC oscillations under DD.

### Diurnal oscillations of acylcarnitines are not strictly coupled to rhythms in locomotor activity, but may correlate with feeding rhythmicity in wildtype flies

**Fig. 5:**
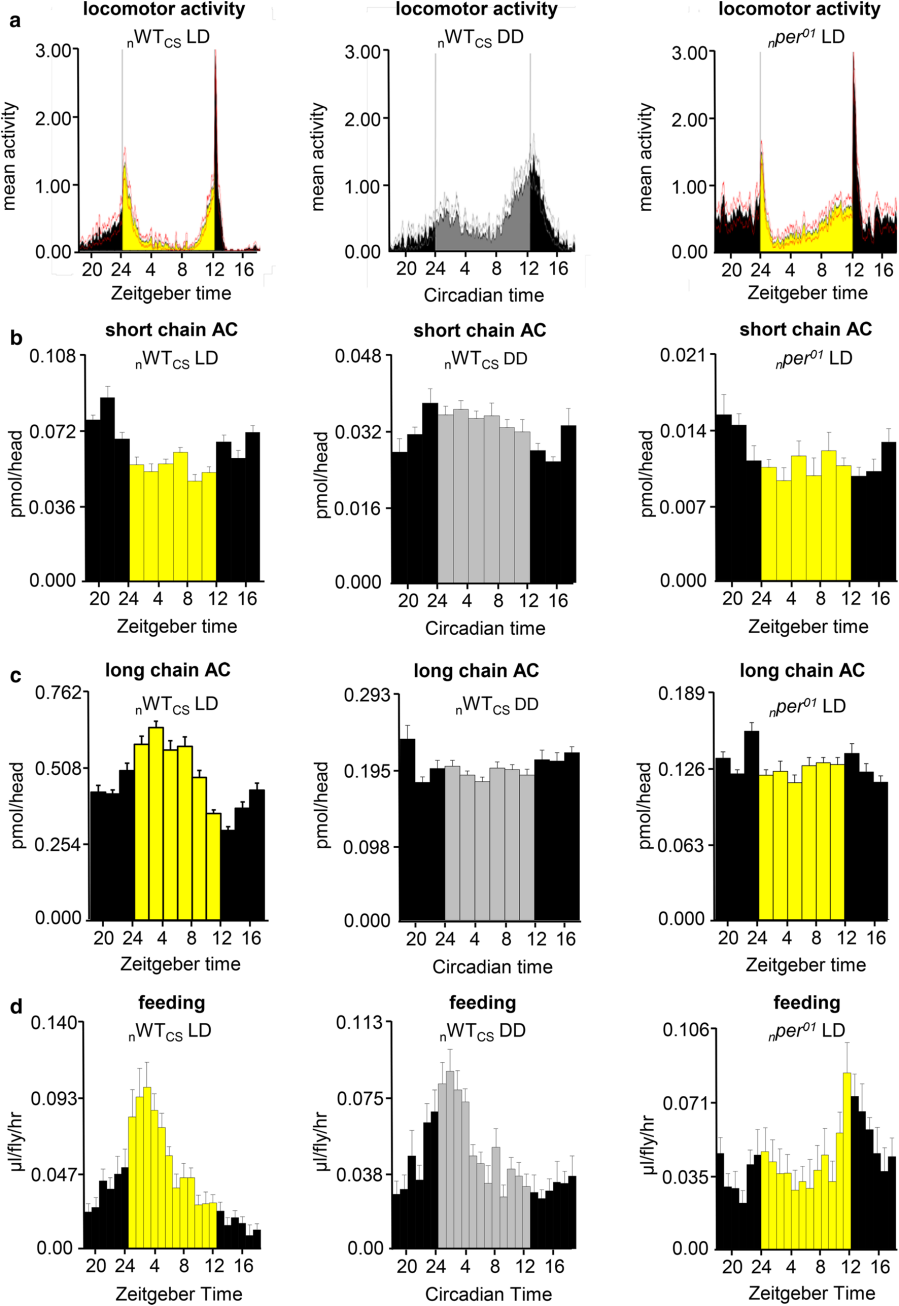
Correlation between locomotor and feeding activity and AC level oscillations in WT_CS_ in LD (left) and DD (middle), and in *per*^*01*^ in LD (right). **a** Locomotor activity, **b** short-chain ACs (3:0, 4:0) in fly heads, **c** long-chain ACs (12:0–18:2) in fly heads, **d** food consumption. Locomotor activity was monitored for 7 days under LD (*n* = 45) and at the first day under DD (*n* = 43). AC levels were determined every 3 hours for three consecutive days under LD and two consecutive days under DD (*n* = 4–7). Food consumption was monitored every hours for three consecutive days (*n* = 5). Data represent mean ± s.e.m

ACs are critical for the transfer of fatty acids into mitochondria, where they undergo ß oxidation utilized for energy production [45]. We, therefore, hypothesized that diurnal oscillation of ACs might correlate with the daily rhythm in energy-consuming locomotor activity, and monitored rest/activity of WT_CS_ and *per*^*01*^ flies. As expected, locomotor activity in WT_CS_ peaked in the morning and evening under LD and remained biphasic even in DD, independent of the genetic background (Fig. 5a). In contrast to these typical biphasic oscillations, ACs showed monophasic oscillations or lost rhythmicity (Fig. 5b, c). Rhythmic levels of ACs, therefore, are not directly correlated with physical activity under LD and DD conditions in wildtype flies, at least under our laboratory conditions which prevent longer flight activity. Hence, it is unlikely that time-dependent variations of AC levels in fly heads and bodies are a direct consequence of physical activity-dependent energy consumption.

In mammals, AC plasma levels vary depending on the feeding (fed or fasted) state [46]. To study whether AC levels in fly heads and bodies can be linked to feeding–fasting cycle, a modified CAFE assay [37] was employed to monitor food consumption in _*n*_*per*^*01*^ and _n_WT_CS_ flies (Fig. 4d). In accordance with the literature [10, 47], feeding activity showed a monophasic oscillation peaking at ZT3 (BH.Q = 10^–35^, AMP.rel = 28%) in _n_WT_CS_ flies under LD. Hence, feeding behavior was in phase with the rhythm of long-chain ACs in _n_WT_CS_ under LD. In _n_WT_CS_ under DD, daily rhythmicity of food consumption remained monophasic (BH.Q = 10^–15^, LAG = ZT0.5, AMP.rel = 21%) similar to the fluctuation of AC(3:0) and AC(4:0) levels as shown in Fig. 5b, d. It thus seems possible that feeding activity is connected to the rhythm of short-chain ACs under DD. In contrast, the oscillations of long-chain ACs appear to be driven by light as they largely disappeared in DD (Fig. 5c, Suppl. Figure 10, Suppl. Table 5) despite continued feeding rhythmicity.

In _*n*_*per*^*01*^ under LD, food consumption was still cycling (BH.Q = 10^–6^, LAG = ZT15.5, AMP.rel = 53%), but the maximum shifted ~ 15 h compared to _n_WT_CS_ (Fig. 4d). In contrary to feeding rhythmicity, daily rhythmicity of short-chain as well as long-chain ACs dampened in _*n*_*per*^*01*^ compared to _n_WT_CS_ (Suppl. Figure 10, Suppl. Table 5). Taken together, this data suggest that food consumption and AC levels are linked in wildtype flies, but become decoupled in *per*^*01*^ clock mutants.

### *per*^*01*^ flies are more sensitive to starvation despite of wildtype-like food intake

The results above suggest that daily variations of AC levels are not directly linked with locomotor activity, but might be associated with feeding behavior. We, therefore, tested whether the different levels of DAGs and ACs between *per*^*01*^ and WT_CS_ flies can be attributed to differences in total mean activity or total food consumption as all three lipid classes are closely linked to energy expenditure and digestion. First, we compared fresh and dry weights between _*n*_*per*^*01*^ and _n_WT_CS_ flies (Fig. 5a). Significant differences in weights between the two genotypes were not detectable (Suppl. Table 2). Thus, altered levels of differing metabolites are unlikely to be attributed to differences in body weight. We next compared total food consumption. Surprisingly, although the temporal feeding profile differed between _*n*_*per*^*01*^ and _n_WT_CS_, total food consumption between the two genotypes in the CAFE assay was similar (Fig. 5d). Taken together, these results indicate that the lower metabolite levels observed in *per*^*01*^ clock mutants can neither be attributed to lower food intake, lower energy stores nor to lower body mass.

**Fig. 6:**
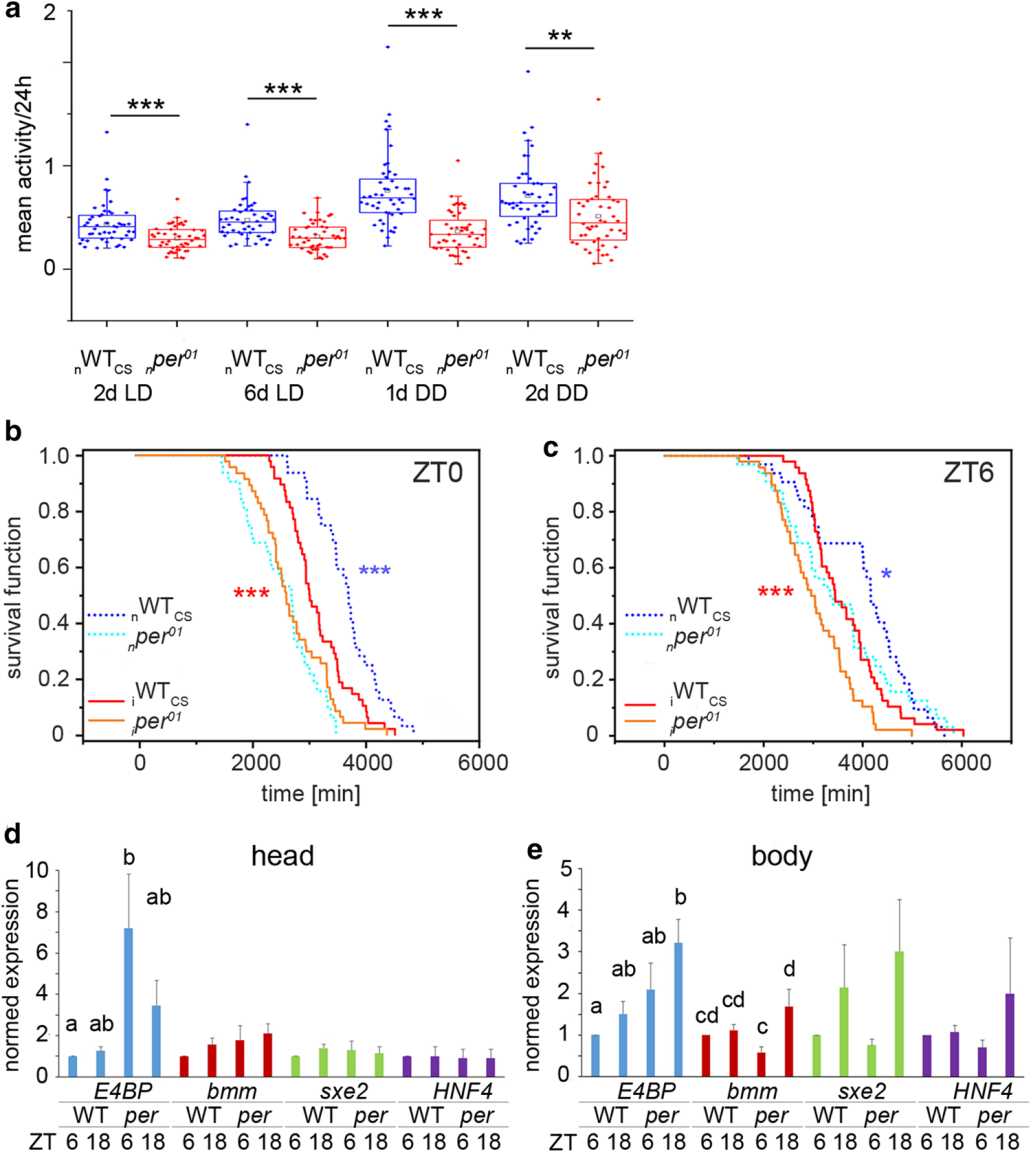
**a** Mean locomotor activity (beam crosses/min) of _n_WT_CS_ (blue box plots) and _n_*per*^*01*^ (red box plots) at day 2 and day 6 in LD, and day 1 and day 2 in DD, n = 48. **b**, **c** Kaplan–Meier survival curves for flies placed under starvation at (**b**) ZT0 or (**c**) ZT6, *n* = 100. Note that flies survived longer when starvation was started at ZT6: Kaplan–Meier estimated mean ± s.e.m.: ZT0: _n_WT_CS_ = 3679.3 ± 97.0, _*n*_*per*^*01*^ = 2548.8 ± 106.0, _i_WT_CS_ = 3162.6 ± 76.8, _*i*_*per*^*01*^ = 2721.0 ± 89.9; ZT 6: _n_WT_CS_ = 3978.6 ± 180.3, _*n*_*per*^*01*^ = 3514.9 ± 204.0, _i_WT_CS_ = 3645.0 ± 110.7, _*i*_*per*^*01*^ = 3043.0 ± 106.5. **d** Normed expression levels of metabolically relevant genes as quantified by RT-qPCR in the head (left) or body (right) of _n_WT_CS_ and _*n*_*per*^*01*^ flies. *n* = 3, *N* = 4. Different letters indicate significant differences (*p* < 0.05). Data in a and d-e are shown as mean ± s.e.m. Asterisks denote statistically significant differences (*p < 0.05, **p < 0.01, ***p < 0.001, *t *test) between the genotypes

Impaired lipid metabolism can lead to starvation and starvation-induced hyperactivity [40, 48]. We, therefore, calculated the total locomotor activity under LD conditions which was significantly higher in _*n*_*per*^*01*^ compared to _n_WT_CS_ flies, but significantly lower in _*i*_*per*^*01*^ flies compared to _i_WT_CS_ (Fig. 6a). The background-independent metabolic phenotype of *per*^*01*^ mutants is thus unlikely to be linked to the general activity level.

As lower levels of ACs and DAGs in bodies of *per*^*01*^ were not correlated with locomotor activity, we hypothesised that *per*^*01*^ flies might behave differently under starvation stress conditions. Therefore, we assayed starvation resistance of *per*^*01*^ and WT_CS_ flies by food-depriving flies starting in the morning (ZT0-1) or afternoon (ZT6-7). Clock mutant flies were significantly more sensitive to starvation (Breslow test, Kaplan–Meier survival function) under both conditions, independent of the genetic background (Fig. 6b, c), and fully consistent with published data [16, 49, 50]. Interestingly, all strains lived longer when starvation started at ZT6 after the morning feeding bout (Fig. 6c) compared to ZT0, at the time when feeding begins (Fig. 6b).

### The metabolic stress marker 4EBP is increased in *per*^*01*^ flies

To obtain first hints which transcriptional metabolic pathways might be affected by *per*^*01*^, we measured mRNA expression of key metabolic genes in whole heads and bodies of _*n*_*per*^*01*^ and _n_WT_CS_ flies in LD with ad libitum access to food, at the same time points (ZT6 and ZT18) as used for metabolite sampling (Fig. 6d, e). Interestingly in light of the increased starvation sensitivity described above, _*n*_*per*^*01*^ flies on normal food showed increased expression of *4EBP* (aka *Thor*, Fig. 6d, e). *4EBP* is a TOR-regulated gene coding for a translation initiation factor binding protein which enhances mitochondrial activity [51] and is upregulated upon environmental stress including starvation [52]. In contrast, we found no consistent and significant differences in expression for sampling times and genotypes for *brummer (bmm)* and *sxe2* (Fig. 6d, e) which encode metabolically relevant lipases [53, 54], further supporting that lipid mobilisation is not affected by the *per*^*01*^ mutation. Also the expression of *Hnf4* encoding a transcription factor which in adult flies is required for glucose homeostasis, oxidative phosphorylation and mitochondrial function [55], was not affected by an impaired clock (Fig. 6e).

## Discussion

### ***per***^***01***^ mutation affects the level of specific lipids independent of the genetic background

A major outcome of our study is the finding that a strong effect of a clock mutation on the head or body metabolome was seen on specific lipids (DAGs, ACs) as well as the amino acid tryptophan, all of which were reduced in bodies of *per*^*01*^ mutants in a background-independent manner. In contrast, an impaired molecular clock in *per*^*01*^ mutants on standard food obviously does not affect general energy stores as carbohydrate and TAG levels were largely unaffected. Moreover, the general level of food intake was not significantly altered between *per*^*01*^ mutants and wildtype flies. We would like to stress that these results are obtained from whole-body clock mutants, in which central and peripheral clocks are likewise affected. Moreover, level differences between different organs may have cancelled each other out in our global analysis. For example, the central clock in the brain appears to oppose that of the fat body [47] and hence more pronounced metabolomic effects might appear if the clock is only perturbed in specific metabolic or neuronal tissues.

DAGs are the transport form of glycerolipids in the insect hemolymph providing fatty acids for acyl-CoA production at the target tissues (see [56]) and hence serve as precursors of ACs which are required for fatty acid transport across the inner mitochondrial membrane. These lipid species were also found to oscillate in a clock- and light-dependent manner, strengthening a functional link between *per* and lipid metabolism.

A recent meta-analysis of available chronometabolomic studies in humans revealed a large variability in detected differential metabolites [17]. Among 9 analysed sleep and circadian studies, only 43 significantly different metabolites were cross replicated in at least 1 circadian and 1 sleep study. Out of these 43, only 12 compounds were found in at least 2 circadian or sleep studies. Interestingly, 3 ACs (C8:0, C12:0, C18:1) and tryptophan are among these 12 compounds. Our finding of significant differences in ACs and tryptophan, confirmed in two different backgrounds, adds further evidence that these compounds are under clock regulation across tissues. Furthermore, as our findings are from the phylogenetically very distant fruit fly, our results suggest that the clock control of these compounds may represent an evolutionarily old and basic feature.

Several *Drosophila* studies quantified levels of storage lipids (TAGs) and reported lower levels in clock mutants compared to wildtype flies [10, 16, 49, 50], while only one study is in line with our results and did not find an effect of various clock mutations including *per*^*01*^ on abdominal fat body TAG levels [15]. A comparison between these and our studies is not straightforward as different techniques, including LC–MS, colorimetric and enzymatic assays, were used to quantify TAGs in *per*^*01*^ or *tim*^*01*^ clock male or female mutant flies raised on different food. A further complication is that our study measured heads and bodies separately, while others measured whole flies. In addition, we did not starve the flies prior to the assay as we wanted to avoid metabolic changes caused by short-term starvation. Thus, guts still contained digested dietary lipids when flies were sampled. TAGs and DAGs can be directly derived from ingested food in the midgut, beside *de novo* biosynthesis in the fat body, oenocytes and digestive system [57]. Since the clock mutants and the wildtype flies consumed similar amounts of food, we assume that the observed differences in metabolite levels are unlikely to be caused by differences in direct dietary lipid absorption. We can, however, not exclude that *per*-dependent metabolic levels of TAGs and DAGs have to any degree been masked at least in fly bodies, the compartment in which nutrient uptake takes place. Unlike previous studies focussing on TAGs [10, 16, 49, 50], we also determined DAG levels, which were found to be lowered in the body of clock mutants. This is a novel finding and it will be interesting to see whether it can be repeated in other clock gene mutants and conditions.

### *per* has an impact on light-driven oscillation of acylcarnitine levels

In our study, the most pronounced diurnal oscillations in wildtype metabolite levels were found for ACs. Remarkably, daily cycling of ACs has also been found in human plasma [6–8], suggesting that this may be a shared metabolic feature between flies and humans. Confirming our observation, recent metabolomic analysis revealed daily rhythms in AC levels in bodies of w^1118^ flies [14]. Under constant darkness, diel rhythm of several AC levels remained in w^1118^ [14], whereas in our study only short-chain ACs continued to oscillate in _n_WT_CS_. Strikingly, similar phase shifts were present between LD and DD in both studies. The loss of diel rhythmicity of long-chain ACs in DD point towards light-driven processes underlying the observed oscillations. As feeding and locomotor activity continued to cycle in DD in _n_WT_CS_, the AC oscillations obviously are independent of both processes and the cause of the light-dependent AC oscillations remain unclear. These processes seem, nevertheless, to depend on *per* activity since the oscillations are abolished or strongly weakened in LD in *per*^*01*^ mutants. Reminiscent of recent studies in mice [58–60], our data thus point towards an interaction of *per* clock gene as well as light in the generation of diel oscillating metabolite levels.

The observed differences in the daily rhythms of short-chain ACs [with exception of AC(2:0)] and long-chain ACs in _n_WT_CS_ under LD might be explainable by their different biosynthetic pathways. Long-chain ACs are synthetized from carnitine and long-chain acyl-CoA by carnitine palmitoyltransferase 1 (CPT1) [45], which levels oscillated in a PER1/2-dependent diel manner in isolated mitochondria in mice [58]. Also the levels of acylcarnitine carrier protein and CPT2 used to transport ACs across the mitochondrial membranes and to release free acyl CoAs for β oxidation show diel cycling in mice [58]. Though data of mitochondrial enzyme cycling are lacking for *Drosophila*, similar mechanisms might underlie the observed oscillations of AC levels. Short-chain ACs are mainly produced from branched-chain amino acids via short-chain acyl-CoA in the mitochondria [61]. Carnitine–acylcarnitine translocase transports back the mitochondrial carnitine and AC(2:0) to the cytosol. As daily rhythm of AC(2:0) was more similar to long-chain ACs than to the other short-chain ACs, it is possible that the substrate of AC(2:0) is acetyl-CoA which is produced in excess during β oxidation.

In this study, AC rhythms were more pronounced in heads than bodies of _n_WT_CS_. As β oxidation in the *Drosophila* CNS is carried out by glia cells [62], we think that the stronger AC oscillation in heads reflects daily changes in the metabolic activity of glia cells which likely contributes more to the overall metabolic signals in heads than in bodies. In the body, the midgut is one potential source contributing to AC oscillations as it is rich in short-chain as well as long-chain ACs. Other potential sources include muscles and fat body, tissues with high mitochondrial activity. Taken together, our findings reveal that time-dependent levels of ACs are lipid-species specific, but must occur in phase across major organs. It is clear, however, that the involved mechanisms remain to be identified.

Besides the observed diel oscillations, we further found that long-chain AC levels were consistently lower in the body of *per*^*01*^ compared to WT_CS_ flies, while AC3:0 levels were consistently higher. The low levels of long-chain ACs are compatible with the idea of reduced CPT activity in *per1/2*^*−/−*^ in mice [58]. Mutation of mouse *Bmal1* also affects ACs levels. For example, levels of AC(2:0) and AC(6:0) were lower in muscles of mice carrying a myocyte-specific disruption of the clock protein BMAL1 [63], while long- and medium-chain fatty acylcarnitines were higher in starved *Bmal1* mice compared to control under DD [64]. This further suggests that ACs represent basal core clock-dependent metabolites. We also note that transcription of *pudgy*, encoding for a long-chain fatty acid CoA-ligase that acts directly upstream of CPT1 in *Drosophila*, has been identified to cycle in transcriptomic studies [65]. This provides further evidence for clock-dependent regulation of mitochondrial activity and a possible link to insulin/FOXO signalling [66]. Interestingly, multifactorial roles of ACs in neuroprotection have been found in humans [67]. It may, therefore, be interesting in the future to test whether the observed AC decrease in *per*^*01*^ mutant flies contributes to the reported accelerated oxidative stress-induced neurodegeneration in *per*^*01*^* sni*^*1*^ flies [68].

### Several lines of evidence point towards altered mitochondrial activity in *per*^*01*^ flies

DAGs and ACs, the metabolites most affected in *per*^*01*^ mutants in our study, are precursors for mitochondrial fatty acid oxidation. Long-chain ACs and carnitine levels can even be used to identify disturbances in mitochondrial metabolism, as their levels are modulated by the ratio of acyl-CoA and CoA which in turn regulates the activity of mitochondrial enzymes [69]. In our study, carnitine/long-chain AC ratios were 1.4–3 times higher in heads and bodies of *per*^*01*^ compared to WT_CS,_ while their absolute levels were down to 0.1–0.8 times. This indicates an impact of the *per* mutation on mitochondrial activity, but speaks against impaired β oxidation which rather should lead to a decrease in the carnitine/long-chain AC ratio. In addition, _n_*per*^*01*^ flies showed significantly upregulated levels of the translational repressor 4EBP which is upregulated upon dietary restriction and likely contributes to increased mitochondrial efficiency [51] and inhibits TAG mobilisation [52]. It seems thus possible that upregulated expression of *4EBP* in the *per*^*01*^ mutants is contributing to the lowered DAG levels in the clock mutant. This upregulation in _n_*per*^*01*^ flies might counteract reduced mitochondrial activity.

The idea of altered mitochondrial function in *per*^*01*^ flies is supported by the finding of decreased AC levels as well as altered mitochondrial function in *dfrm1* flies, a *Drosophila* model for fragile-X syndrome [70]. In line with our findings, decreased AC levels in *dmfr1* flies are accompanied by decreased TAG and DAG levels, altered circadian rhythms, hypersensitivity to starvation, and wildtype- like protein content and body weight. Altered mitochondrial activity in *dfrm1* was demonstrated through elevated levels of maximum electron transport system in isolated mitochondria and marked mitochondrial abnormalities in flight muscles. While altered mitochondrial activity in *per*^*01*^ flies has already been suggested by increased mitochondrial hydroperoxyde levels [12] and evidence for a tight circadian control of mitochondrial dynamics and functions is steadily growing in mammals [71, 72], it is clear that further and especially mechanistic studies are required to corroborate the idea of an altered mitochondrial activity in *Drosophila*. Notwithstanding, our results now provide a basis to employ the genetic power of *Drosophila* to identify the still largely unknown mechanisms underlying the circadian control of metabolism.

## Electronic supplementary material

Below is the link to the electronic supplementary material.

Supplementary file1 (XLSX 102 kb)

Supplementary file2 (PDF 8913 kb)
