# Supplementary material for: Loss of function in the Drosophila clock gene period results in altered intermediary lipid metabolism and increased susceptibility to starvation
Source: Cell Mol Life Sci. 2020 Jan 20;77(23):4939–56. doi: 10.1007/s00018-019-03441-6 (PMC7658074; doi:10.1007/s00018-019-03441-6)
Supplement: Supplementary file 2 — Supplementary file2 (PDF 8913 kb) [file 18_2019_3441_MOESM2_ESM.pdf]

Total PEs

a

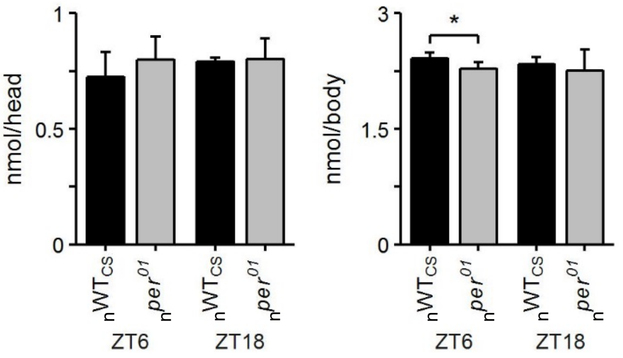

Total PCs

c

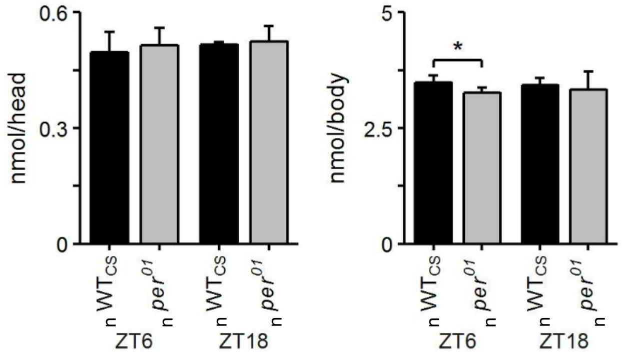

b

Head

c \ db	0	1	2	3	4	5
26	1.3<br>1.2					
28	**1.9**<br>**3.0**	1.1<br>1.0				
30	1.0<br>**1.2**	1.2<br>**1.2**				
31		0.8<br>1.0				
32	0.9<br>**0.9**	1.0<br>0.9	1.1<br>1.1	1.0<br>**1.2**		
33		0.9<br>**0.9**	1.0<br>1.1			
34		1.0<br>1.0	**1.2**<br>1.1	1.1<br>1.1	0.9<br>0.9	
36		1.0<br>0.9	**1.2**<br>1.0	1.1<br>1.0	1.2<br>1.1	1.1<br>1.0
38		0.9<br>**0.9**	1.2<br>1.0			
40						

Body

c \ db	0	1	2	3	4	5
26	**1.2**<br>1.1					
28	**1.3**<br>**1.4**	**1.3**<br>1.2				
30	**1.1**<br>1.4	**1.2**<br>**1.3**	1.0<br>1.0			
31		**0.9**<br>**0.8**				
32	1.0<br>1.1	**0.9**<br>0.9	**0.9**<br>1.0	**0.8**<br>**1.3**		
33		**0.7**<br>**0.7**	**0.8**<br>**0.8**			
34		**0.8**<br>**0.8**	**1.0**<br>1.0	1.0<br>1.0	**0.8**<br>1.0	
36		**0.8**<br>**0.9**	1.0<br>0.9	1.0<br>1.0	1.0<br>1.1	**0.8**<br>1.0
38		**0.7**<br>**0.8**	**0.9**<br>0.9			
40			**0.7**<br>**0.8**			

d

Head

c \ db	0	1	2	3	4	5	6
30	**1.2**<br>**1.2**	1.0<br>1.1	1.1<br>1.1				
32	1.1<br>1.0	0.9<br>**0.9**	1.0<br>1.0	0.9<br>1.0			
34		1.0<br>**0.9**	**1.2**<br>**1.1**	0.9<br>1.0	**0.8**<br>**0.9**		
35			1.0<br>0.9		n<br>n		
36		1.1<br>1.0	**1.2**<br>**1.1**	**1.1**<br>1.0	1.0<br>1.0	1.0<br>1.0	**0.8**<br>**0.8**
37							
38							1.0<br>1.0

Body

c \ db	0	1	2	3	4	5	6
30	**1.5**<br>**1.8**	**1.2**<br>**1.2**	**1.2**<br>1.1				
32	**1.3**<br>**1.4**	**1.1**<br>1.1	**0.9**<br>1.0	**0.9**<br>1.2			
34		**1.1**<br>1.1	1.0<br>1.0	**0.9**<br>**0.9**	**0.7**<br>**0.8**		
35			**0.7**<br>**0.7**		**0.5**<br>**0.5**		
36		1.0<br>1.0	1.0<br>1.0	1.0<br>1.0	**0.8**<br>**0.9**	**0.6**<br>**0.8**	**0.6**<br>0.9
37			**0.4**<br>**0.6**				
38							0.9<br>0.9

Supplementary Figure 1: Profiling of PEs and PCs in *per*^{01} and WT_{CS}. Total levels of PEs (a) and PCs (b) were determined in heads (left plots) and bodies (right plot) of $_{n}WT_{CS}$ (black bars) and $_{n}$*per*^{01} (grey bars) flies sampled at ZT6 and ZT18. Fold differences of PE (c) and PC (d) levels, characterized by total number of carbons and total number of double bonds of the acyl chains, were calculated in heads (left panel) and bodies (right panel) of *per*^{01} versus WT_{CS} sampled at ZT6 (top of the cell) and ZT18 (bottom of the cell). Statistical significant different lipid ratios ($p<0.05$) are indicated by bold numbers. Abbreviations: C: number of carbon atoms of the acyls, DB: number of double bonds of the acyls. Data represent means ± SD, n=7

Ratio of $_{n}per^{01}$ and $_{n}WT_{CS}$

Head

c \ db	0	1	2	3	4	5
34	**0.6** / **0.4**					
36	**0.6** / **0.4**					
37	**0.5** / **0.3**					
38	**0.6** / **0.5**	**0.6** / **0.4**				
39	**0.5** / **0.4**	**0.6** / **0.4**				
40	**0.8** / **0.7**	**0.7** / **0.5**	**0.8** / **0.6**			
41	**0.7** / **0.5**	**0.7** / **0.5**	**0.7** / **0.4**			
42	0.9 / **0.7**	0.9 / **0.7**	**0.6** / **0.6**	1.0 / **0.7**		
43	**0.7** / **0.6**	**0.7** / **0.6**	0.8 / **0.5**	n.d.		
44	**0.7** / **0.4**	0.9 / **0.8**	**0.7** / **0.6**	**0.5** / **0.4**	**0.7** / **0.5**	
45		**0.7** / **0.6**	**0.8** / **0.6**	0.8 / **0.6**		
46	0.8 / **0.8**	**0.8** / **0.6**	1.0 / **0.7**	**0.7** / **0.6**	0.9 / 0.7	
47		**0.7** / **0.6**	**0.8** / **0.6**	0.9 / **0.6**		
48		0.8 / **0.6**	1.0 / **0.7**	**0.7** / **0.5**	0.9 / **0.6**	
49		0.9 / **0.6**	0.9 / **0.6**	0.9 / **0.6**		
50		**0.7** / **0.7**	**0.6** / **0.4**	1.0 / **0.6**	1.3 / 0.9	**1.6** / 1.2
51		0.9 / **0.6**	1.1 / **0.7**	1.0 / **0.6**		
52			0.8 / **0.5**	**1.3** / 0.9	1.1 / 0.8	**1.4** / 0.9
53		**0.7** / **0.5**	1.0 / **0.7**	1.2 / 0.8		
54						
55		0.8 / **0.6**	1.0 / 0.9	1.3 / 1.1		
56		1.0 / **0.6**		1.1 / **0.6**		
57		1.1 / 0.8	1.2 / 1.0	1.3 / 1.1		
58		1.1 / **0.6**	1.2 / **0.5**	1.1 / **0.5**	1.0 / **0.5**	

Body

c \ db	0	1	2	3	4	5
34						
36	1.1 / 1.1					
37	1.0 / 1.1					
38	1.0 / 1.1	1.0 / 1.0				
39	1.0 / 1.2	0.9 / 0.9				
40	1.0 / 1.0	1.0 / 1.0	**0.8** / **0.8**			
41	1.1 / 1.3	1.0 / 1.1	0.9 / 0.9			
42	1.0 / 1.0	0.9 / 1.0	**0.7** / 0.9	0.9 / 0.8		
43	1.1 / **1.3**	1.0 / 1.2	1.0 / 1.1	**1.5** / **1.5**		
44	1.0 / 0.9	**0.9** / 0.9	**0.8** / 0.9	**0.6** / **0.8**	0.9 / 0.8	
45		0.9 / 1.0	0.9 / 1.1	0.9 / 1.1		
46	1.0 / 1.1	**0.9** / 0.9	1.0 / 0.9	**0.7** / **0.7**	0.9 / 0.9	
47		0.9 / 1.0	0.9 / 1.0	0.9 / 1.1		
48		1.0 / 1.0	0.9 / **0.8**	**0.8** / **0.7**	**0.6** / **0.5**	
49		1.0 / 1.1	1.0 / 0.9	1.0 / 1.0		
50		0.9 / 1.0	**0.8** / **0.7**	1.1 / 0.9	1.2 / 1.0	0.9 / 1.0
51		0.9 / 1.0	0.9 / 0.9	0.9 / **0.8**		
52		0.9 / 0.9	1.0 / 0.8	**1.2** / 1.1	1.0 / 0.9	0.9 / 1.0
53		**0.7** / **0.7**	0.9 / 1.0	**0.7** / **0.7**		
54			**0.8** / **0.8**	**0.8** / 1.0		
55		**0.7** / **0.8**	1.1 / 1.3	1.0 / **1.4**		
56		**0.9** / 0.9		0.9 / 0.8		
57		**0.6** / 0.8	1.1 / 1.3	**1.3** / **1.6**		
58		0.9 / 0.9	1.0 / 0.9	1.1 / 1.0	1.1 / 1.0	

Ratio of $_{i}per^{01}$ and $_{i}WT_{CS}$

Head

c \ db	0	1	2	3	4	5
34	**0.4**					
36	0.8					
37	0.9					
38	1.0	1.1				
39	1.0	1.0				
40	1.0	1.1	1.2			
41	1.0	1.0	1.1			
42	1.0	0.9	1.1	1.3		
43	0.9	0.9	1.0			
44	0.8	0.9	1.0	1.1		
45		0.8	0.9	1.2		
46	**0.6**	0.9	1.0	1.1	1.2	
47		**0.8**	0.9	1.1		
48	0.6	0.9	1.1	1.2	1.3	
49		1.8	0.9	1.1		
50		0.8	1.1	1.3	**1.4**	1.4
51			nd	nd		
52		0.9	1.2	1.3	**1.7**	nd
53			0.6	nd	nd	
54		nd	0.8	**1.3**		nd
55			1.0	**1.3**		
56		nd	**0.6**	0.8		nd
57			1.2			
58			0.6	0.5		

Body

c \ db	0	1	2	3	4	5
34	1.0					
36	0.9					
37	1.1					
38	1.0	1.0				
39	1.2	1.1				
40	1.0	1.0	1.1			
41	1.1	1.1	1.1			
42	1.0	1.0	1.1	**1.3**		
43	1.0	1.0	1.1			
44	0.9	0.9	1.1	**1.2**		
45		0.9	1.1	**1.3**		
46	0.8	0.9	1.1	**1.2**	**1.3**	
47		0.9	1.1	1.1		
48	0.7	**0.9**	1.0	**1.4**	**1.3**	
49		0.9	1.0	**1.1**		
50		0.7	1.0	**1.3**	**1.8**	**2.7**
51			0.9	**1.3**		
52		0.8	0.9	1.1	**1.4**	**1.7**
53			0.9	1.0		
54		**0.7**	**0.8**	1.0	1.1	1.0
55			0.9	1.0		
56		**0.8**	**0.5**	**0.7**		nd
57			0.9			
58			**0.7**	**0.6**		

Supplementary Figure 2: Profiling of lipid species of TAGs in *per*^{01} and WT_{CS}. Fold difference in levels of TAG levels, characterized by total number of carbons and total number of double bonds of the acyl chains, were calculated in heads (left) and bodies (right) of $_{n}per^{01}$ versus $_{n}WT_{CS}$ and of $_{i}per^{01}$ versus $_{i}WT_{CS}$ sampled at ZT6 (top of the cell) and ZT18 (bottom of the cell). Isogenic flies were sampled only at ZT6. Statistical significant different lipid ratios ($p<0.05$) are indicated by bold numbers. Color scheme of heat map: fold difference of 3 (the highest) is dark red, 1 (equal) is white and 0.3 (the lowest) is dark blue. Abbreviations: C: number of carbon atoms of fatty acyls, DB: number of double bonds of fatty acyls, nd: non-detected. Data represent means ± SD, n=7 for the non-isogenic lines an n=4 for isogenic lines

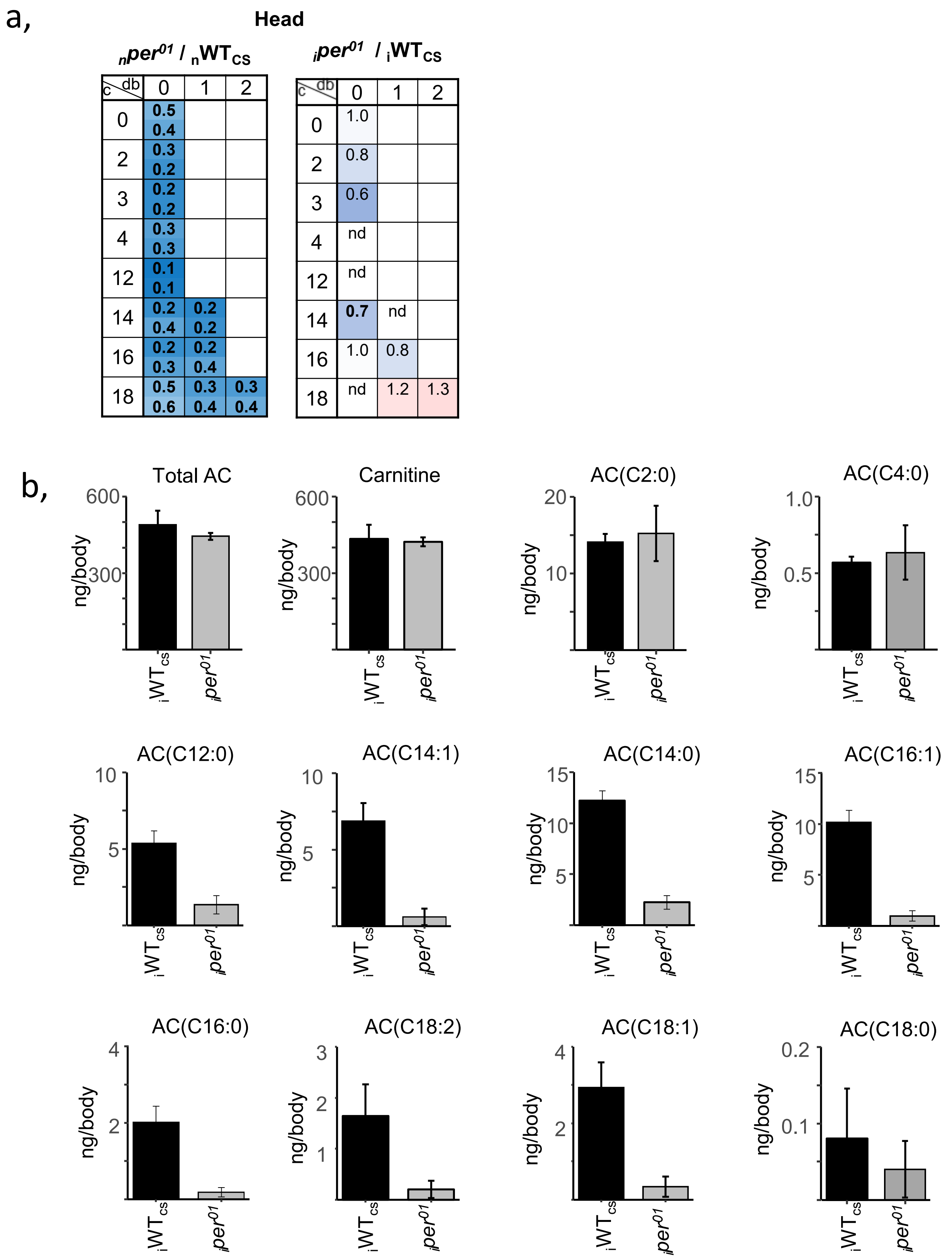

Supplementary Figure 3: Profiling of lipid species of ACs in *per*^{01} and WT_{CS}. (a) Fold difference in levels of AC levels, characterized by total number of carbons and total number of double bonds of the acyl chain, were calculated in heads of $_{n}$*per*^{01} versus $_{n}WT_{CS}$ and of $_{i}$*per*^{01} versus $_{i}WT_{CS}$ sampled at ZT6 (top of the cell) and ZT18 (bottom of the cell). Isogenic flies were sampled only at ZT6. Statistical significant different lipid ratios ($p<0.05$) are indicated by bold numbers. Color scheme of heat map: fold difference of 3 (the highest) is dark red, 1 (equal) is white and 0.3 (the lowest) is dark blue. (b) determined levels of ACs in bodies of of $_{i}$*per*^{01} versus $_{i}WT_{CS}$ at ZT6. Abbreviations: C: number of carbon atoms of fatty acyls, DB: number of double bonds of fatty acyls, nd: non-detected. Data represent means ± SD, n=7 for the non-isogenic lines an n=4 for isogenic lines

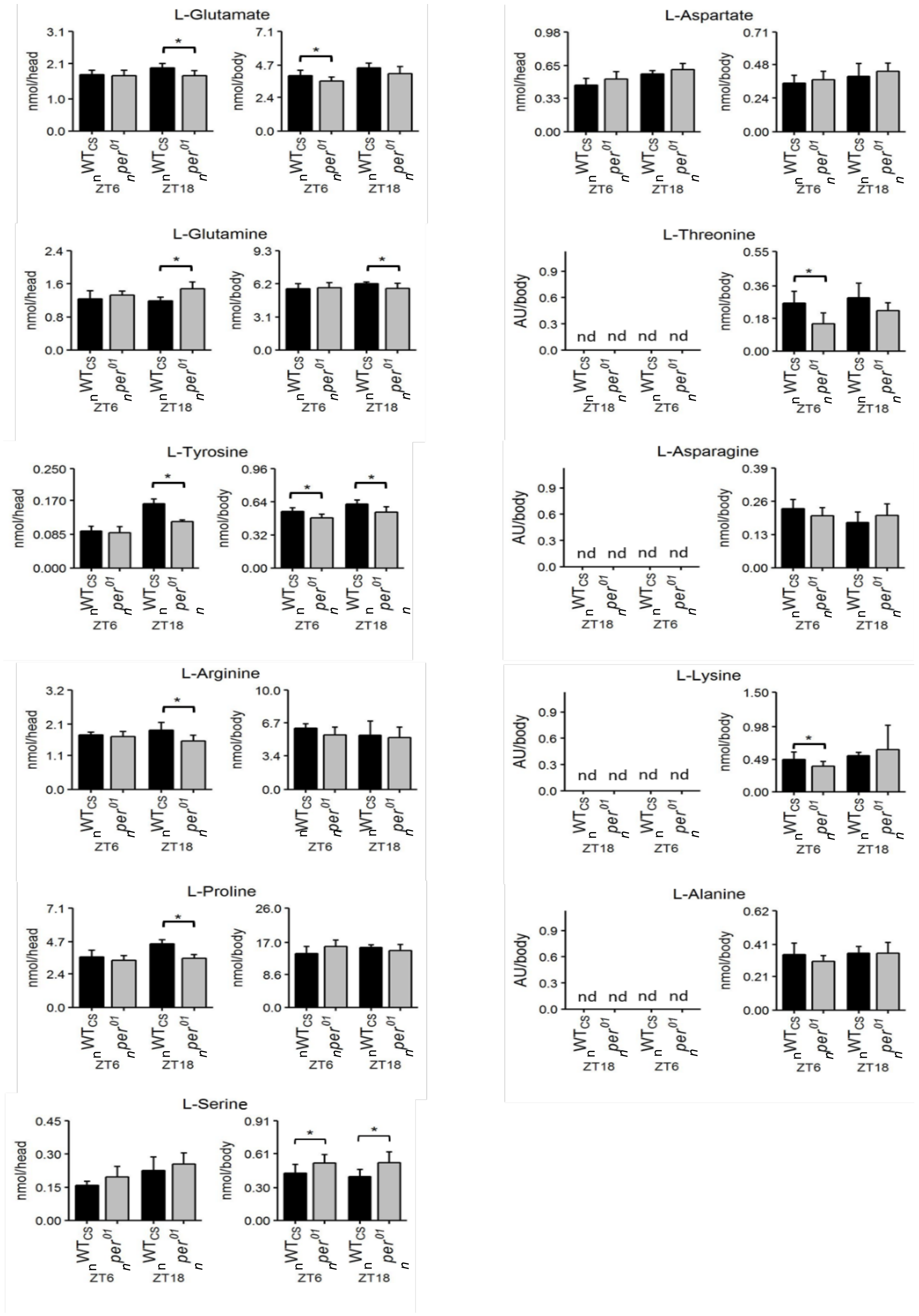

Supplementary Figure 4: Levels of non-essential amino acids in non-isogenic *per*^{01} and WT_{CS}. Levels of glutamate, glutamine, tyrosine, arginine, proline, serine, aspartate, threonine, asparagine, lysine and alanine were determined in heads (left plots) and bodies (right plots) of $_{n}WT_{CS}$ (black bars) and $_{n}$*per*^{01} (grey bars) flies sampled at ZT6 and ZT18. Asterisks denote statistically significant differences ($p < 0.05$) between the two genotypes. Data represent means ± SD, n=5

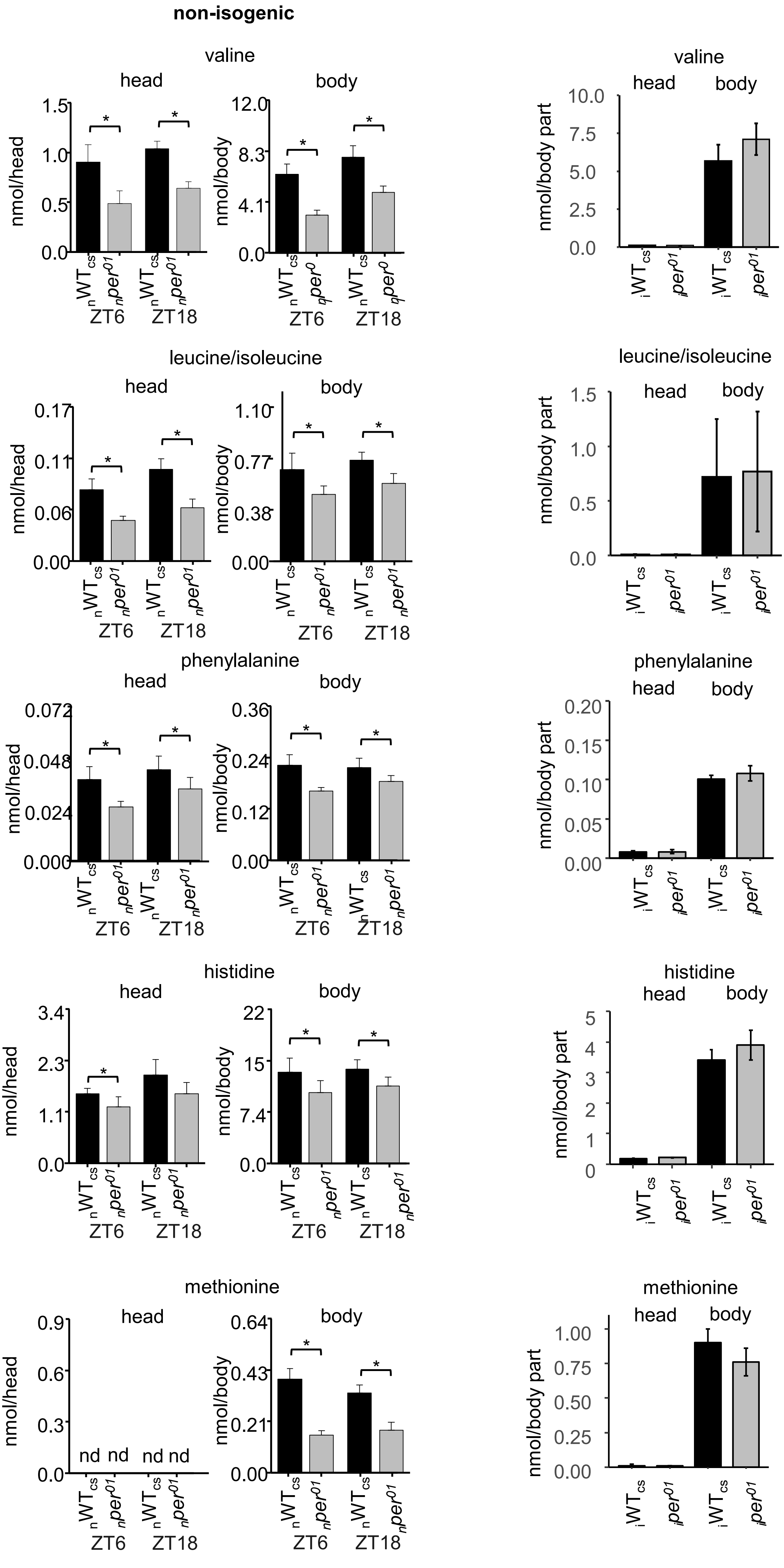

Suppl. Fig 5: Lower levels of essential amino acids in non-isogenic *per*^{01} compared to WT_{CS} but not in the isogenic flies. Total amount of valine, leucine/isoleucine, phenylalanine, histidine, methionine and tryptophan were determined in heads (left plots) and bodies (right plots) of non-isogenic (a) and isogenic (b)WT_{CS} (black bars) and *per*^{01} (grey bars) flies at ZT6 and ZT18. Isogenic flies were sampled only at ZT6. Asterisks denote statistically significant differences ($p < 0.05$, t-test) between the two genotypes. Data represents mean ± SD, n=5. Abbreviation: nd: not-detected

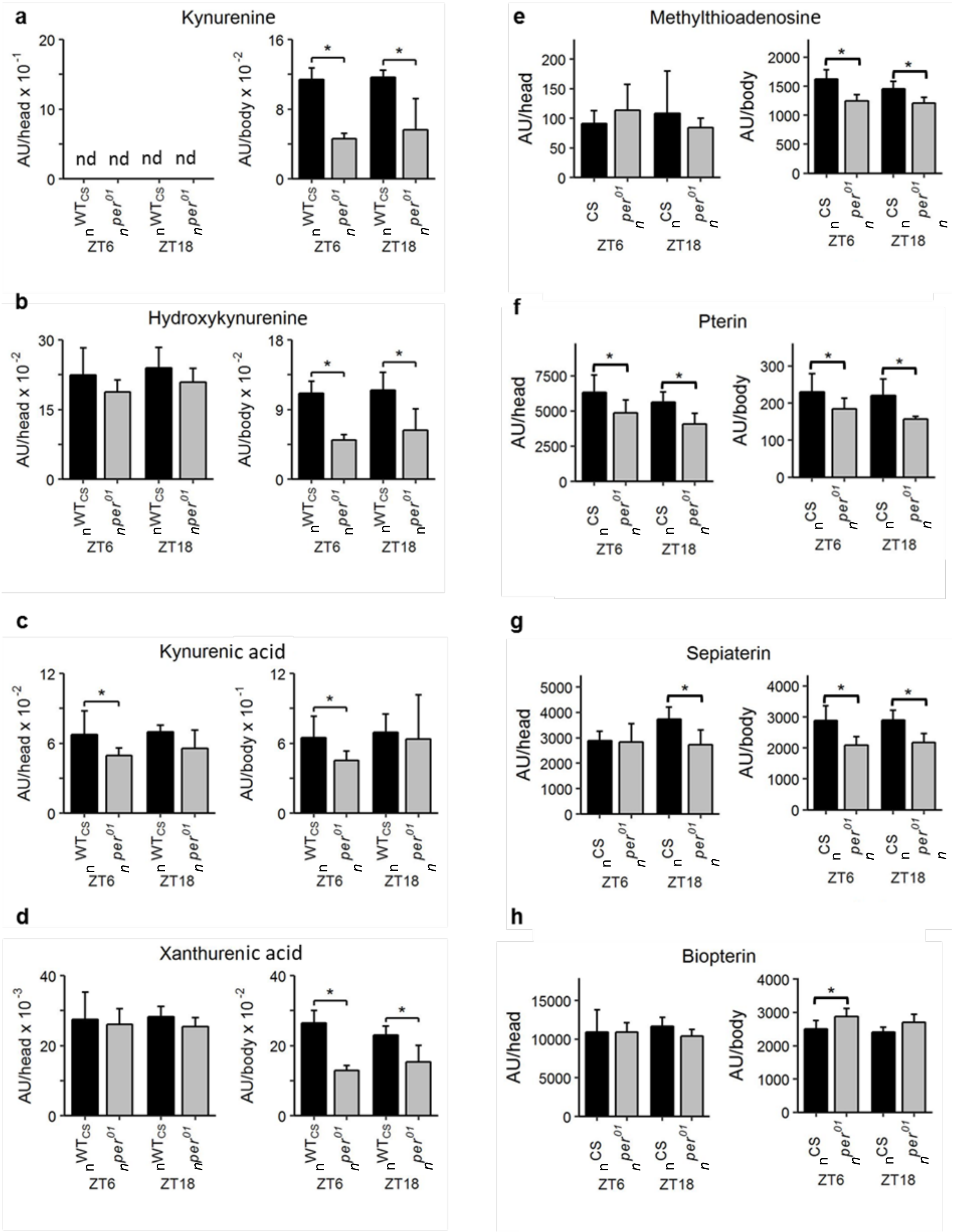

Suppl. Figure 6: Levels of kynurenines, methylthioadenosin and pterinates in $_{n}$*per*^{01} and $_{n}$WT$_{CS}$. Levels of kynurenine (a), hydroxykynurenine (b), kynurenic acid (c), xanthurenic acid (d), methylthioadenosin (e), pterin (f), sepiapterin (g) and biopterin (h) were determined in heads (left plots) and bodies (right plots) of WT$_{CS}$ (black bars) and *per*^{01} (grey bars) flies sampled at ZT6 and ZT18. Asterisks denote statistically significant differences ($p < 0.05$) between the two genotypes. Data represent means ± SD, n=5

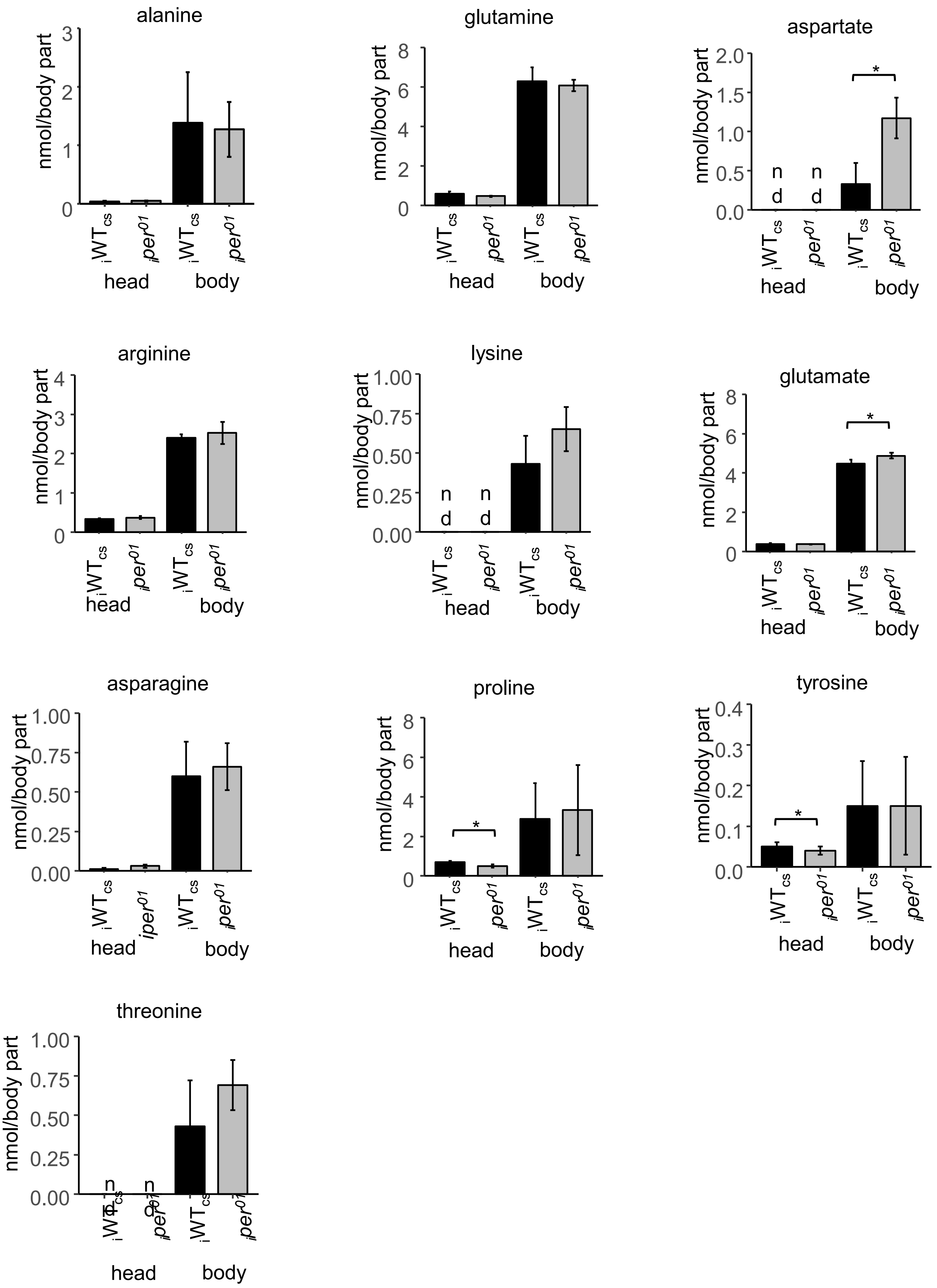

Supplementary Figure 7: Levels of non-essential amino acids in non-isogenic *per*^{01} and WT_{CS}. Levels of alanine, glutamine, aspartate, arginine, lysine, glutamate, asparagine, proline, tyrosine, threonine were determined in heads (left) and bodies (right) of $_{i}WT_{CS}$ (black bars) and $_{i}$*per*^{01} (grey bars) flies sampled at ZT6. Asterisks denote statistically significant differences ($p < 0.05$) between the two genotypes. Data represent means ± SD, n=5.

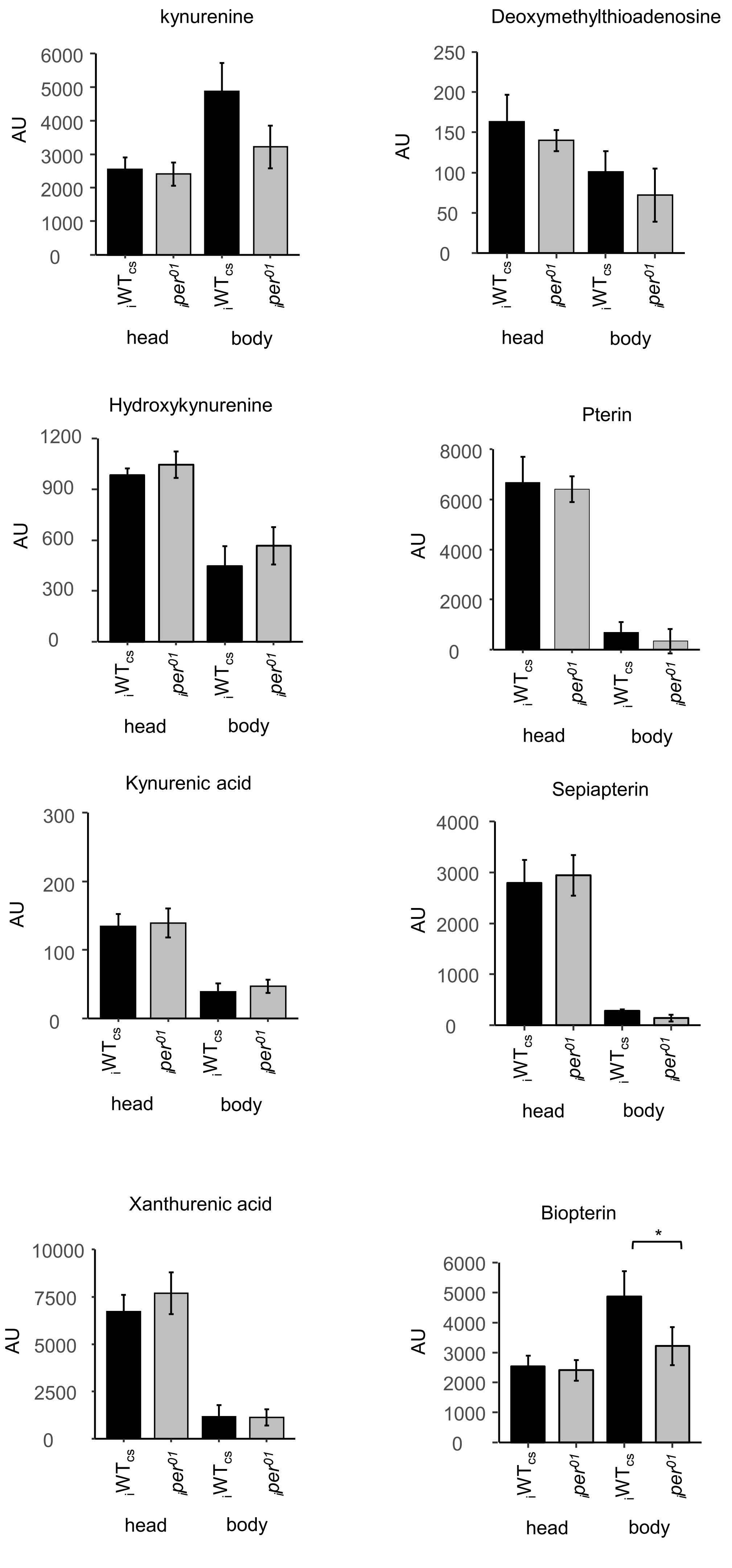

Suppl. Fig. 8: Levels of kynurenines, methylthioadenosin and pterinates in $_{i}$*per^{01}* and $_{i}$WT$_{CS}$. Levels of kynurenine, hydroxykynurenine, kynurenic acid, xanthurenic acid, methylthioadenosin, pterin, sepiapterin and biopterin were determined in heads (left plots) and bodies (right plots) of WT$_{CS}$ (black bars) and *per^{01}* (grey bars) flies sampled at ZT6. Asterisks denote statistically significant differences ($p < 0.05$) between the two genotypes. Data represent means ± SD, n=5

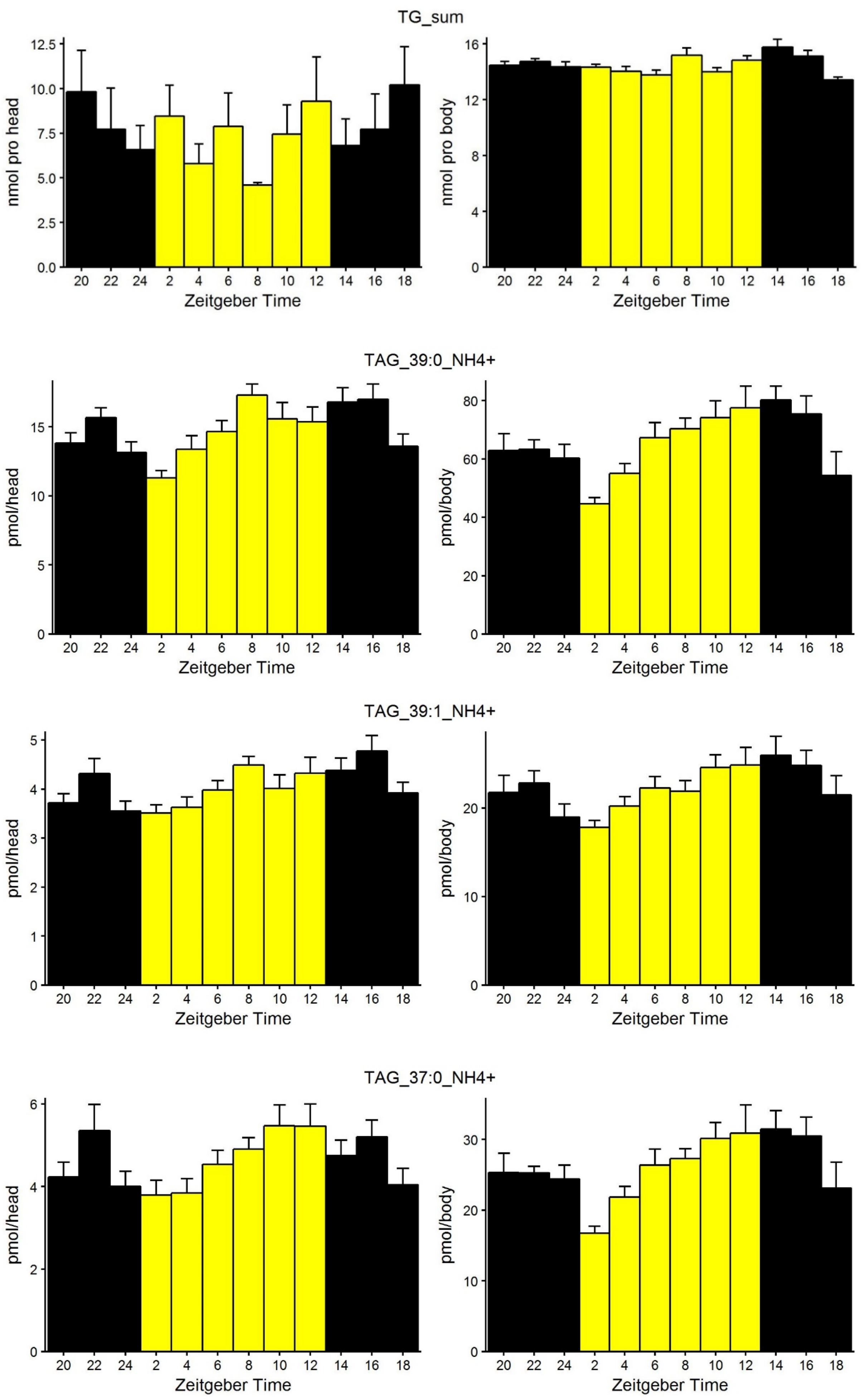

Suppl. Fig. 9 (continued) Daily variations of TAGs and DAGs in WT_{CS} under LD

TAG_40:0_NH4+

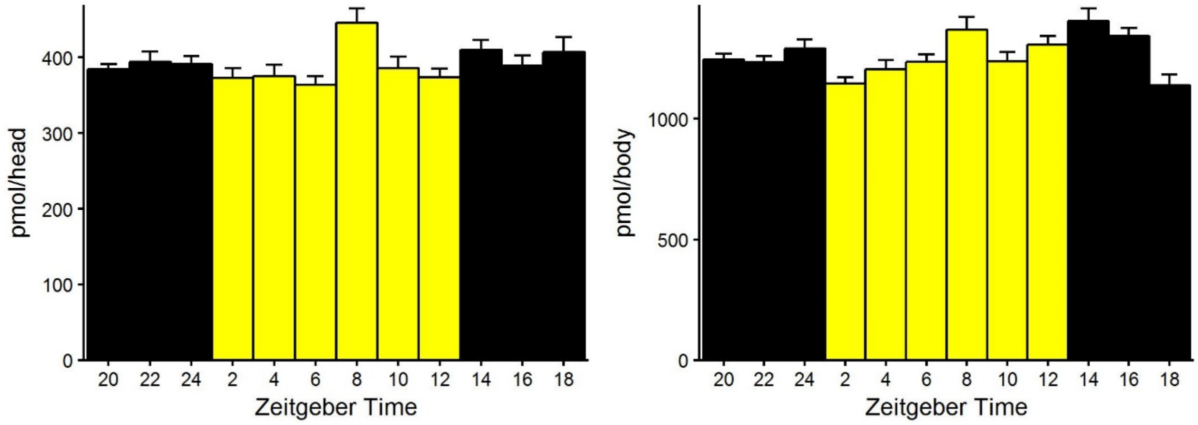

TAG_41:0_NH4+

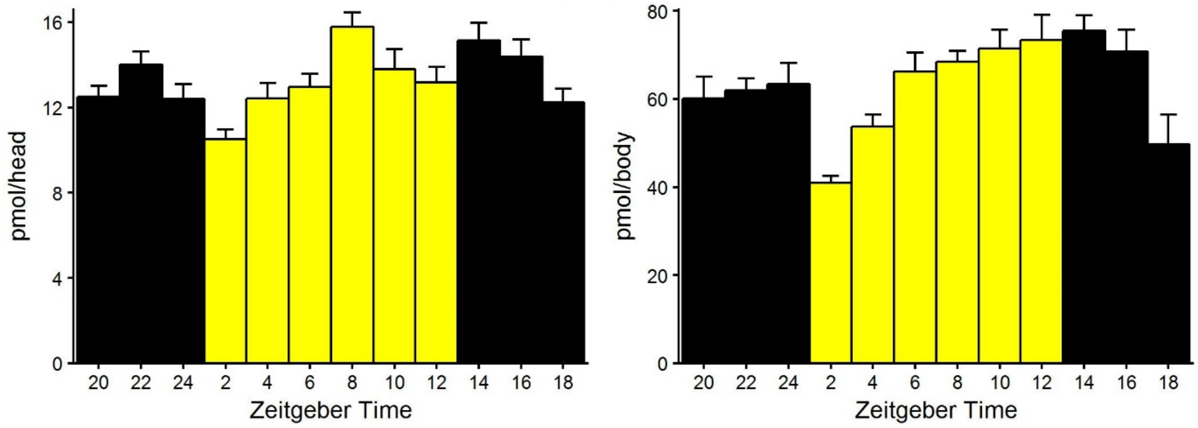

TAG_41:1_NH4+

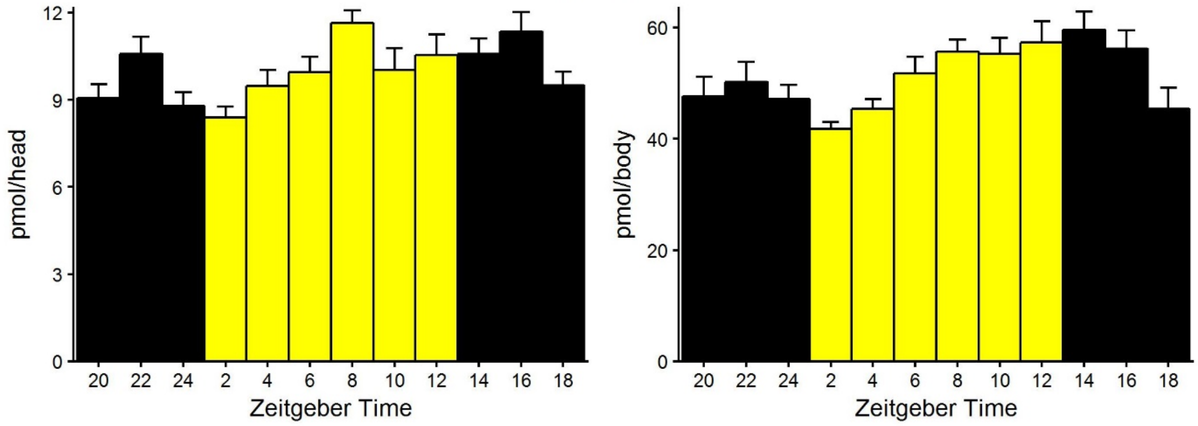

TAG_42:0_NH4+

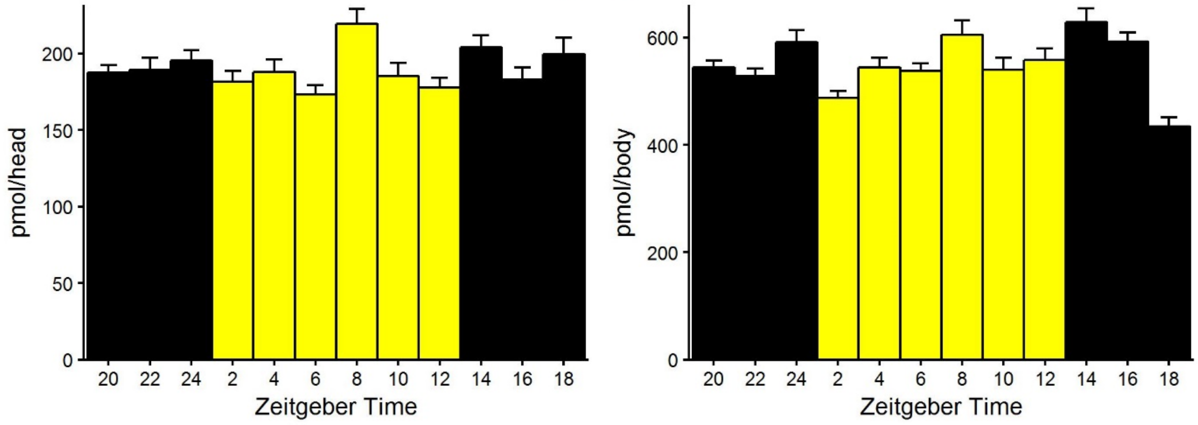

Suppl. Fig. 9 (continued) Daily variations of TAGs and DAGs in WT_{CS} under LD

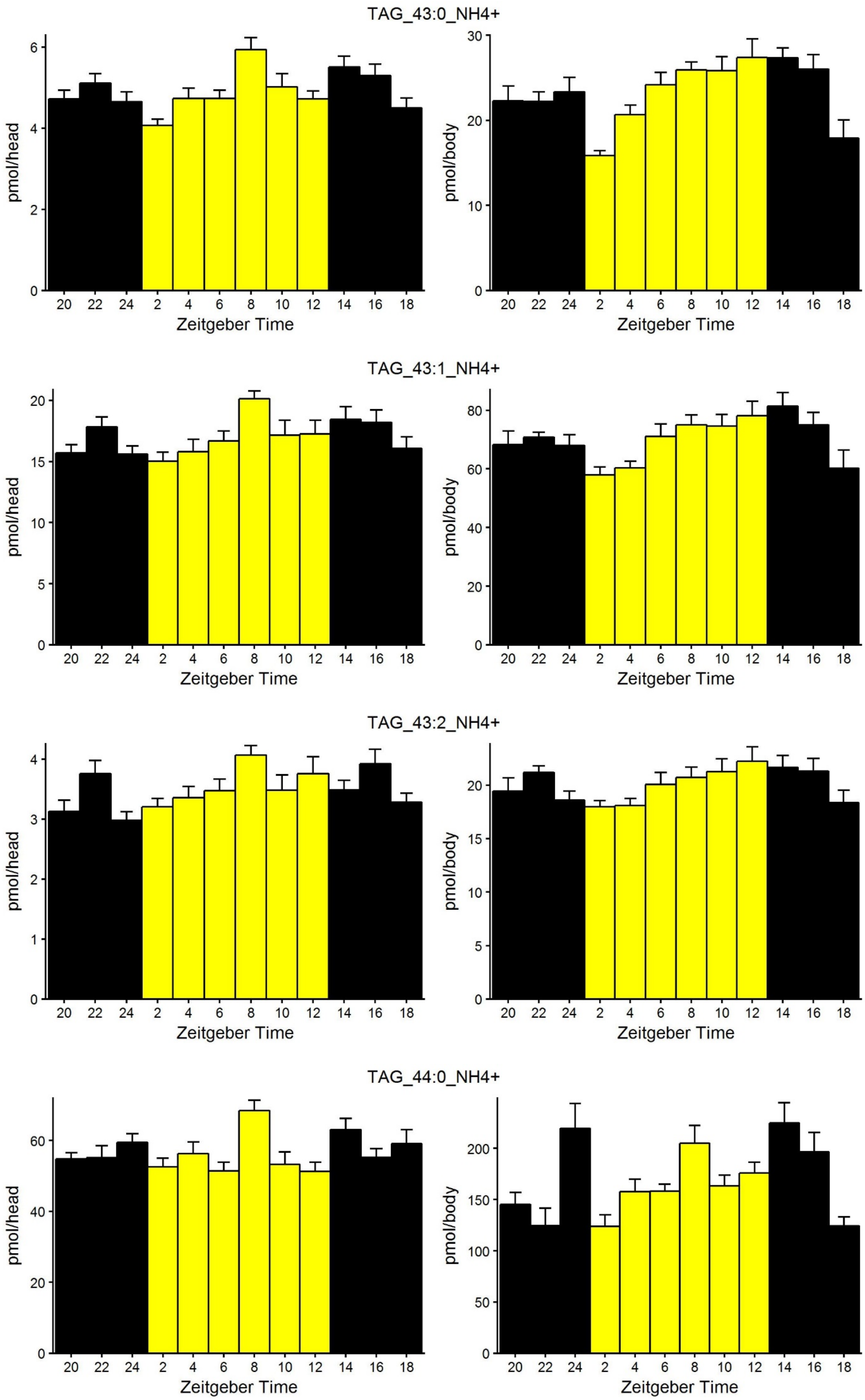

Suppl. Fig. 9 (continued) Daily variations of TAGs and DAGs in WT_{CS} under LD

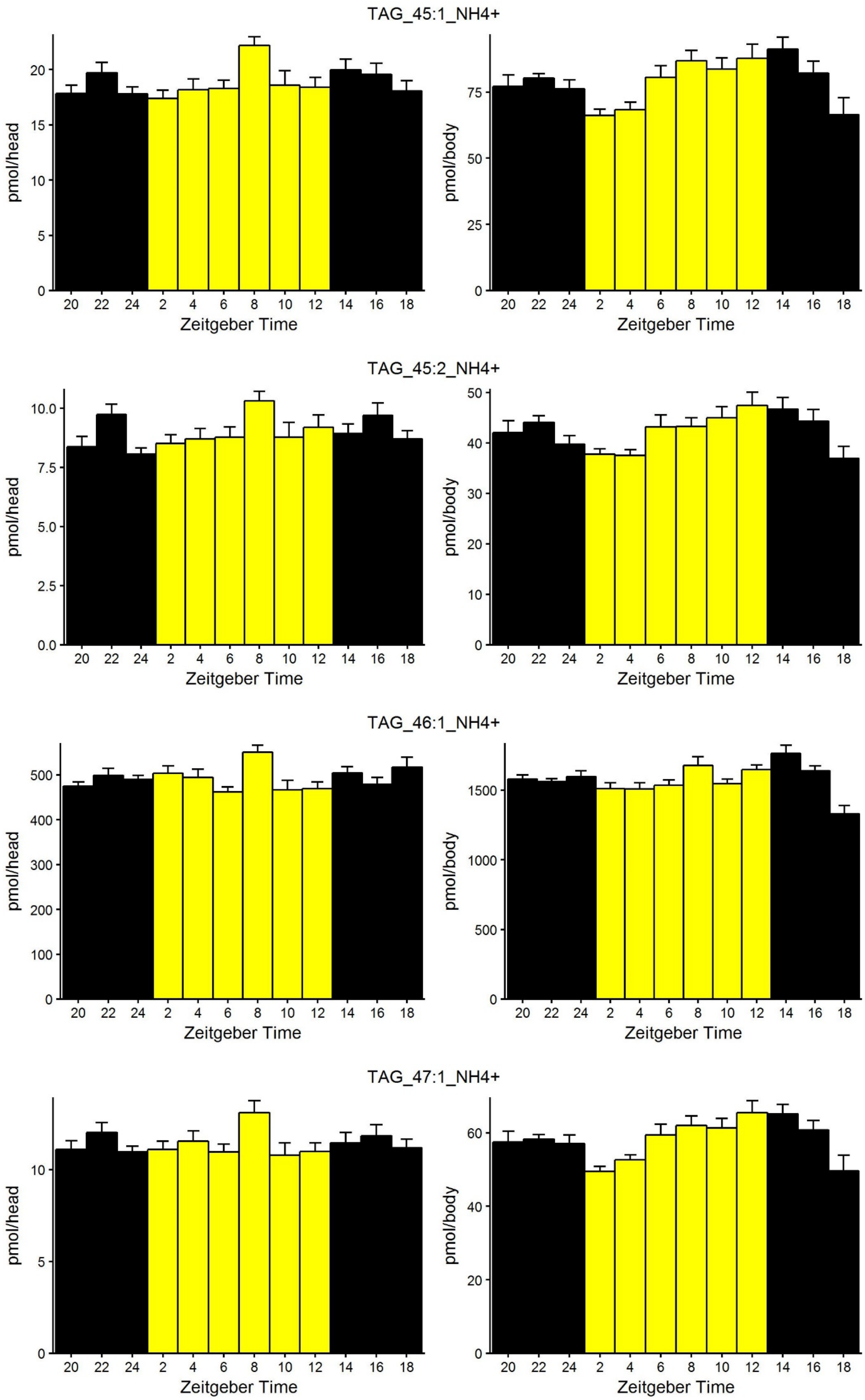

Suppl. Fig. 9 (continued) Daily variations of TAGs and DAGs in WT_{CS} under LD

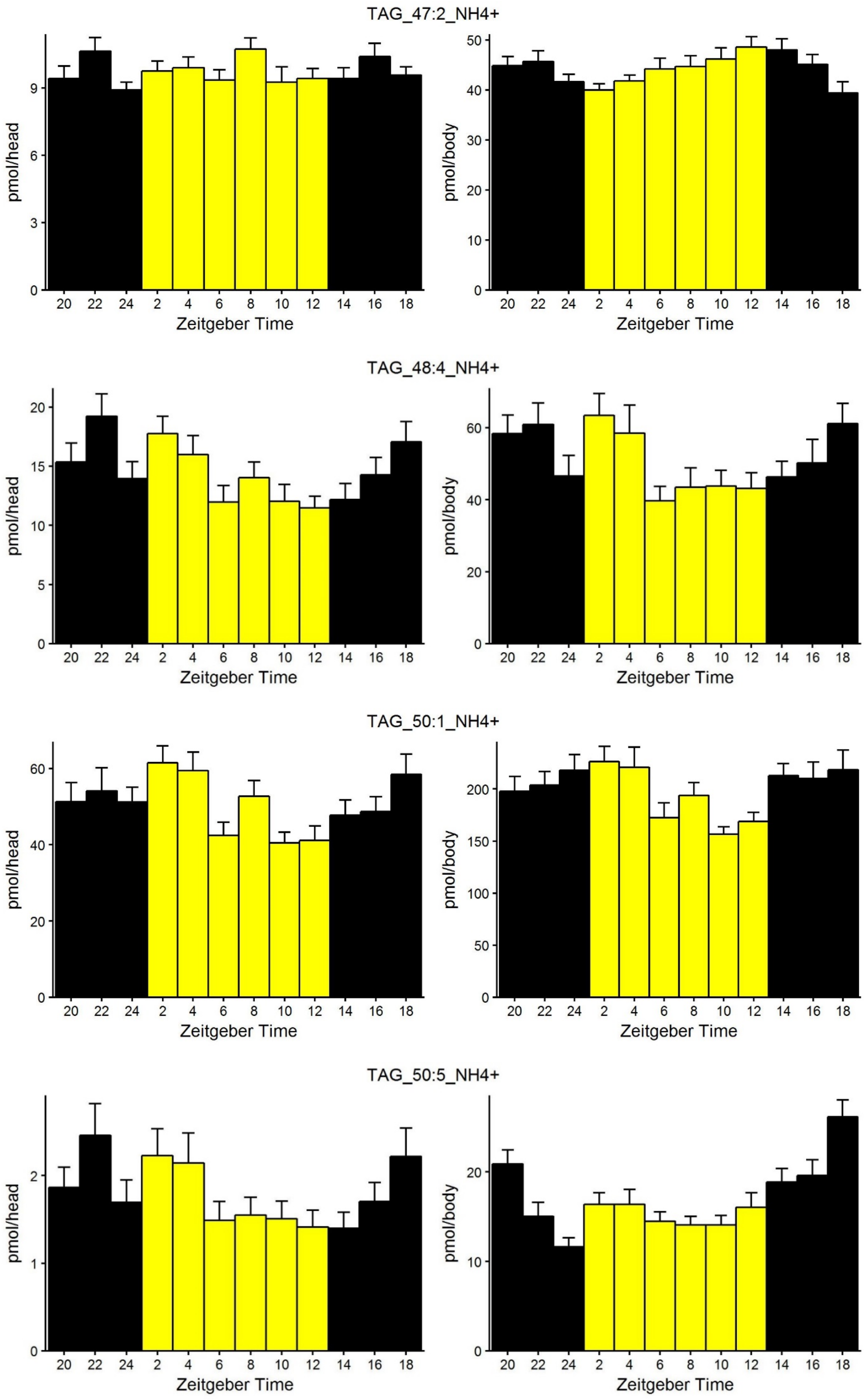

Suppl. Fig. 9 (continued) Daily variations of TAGs and DAGs in WT_{CS} under LD

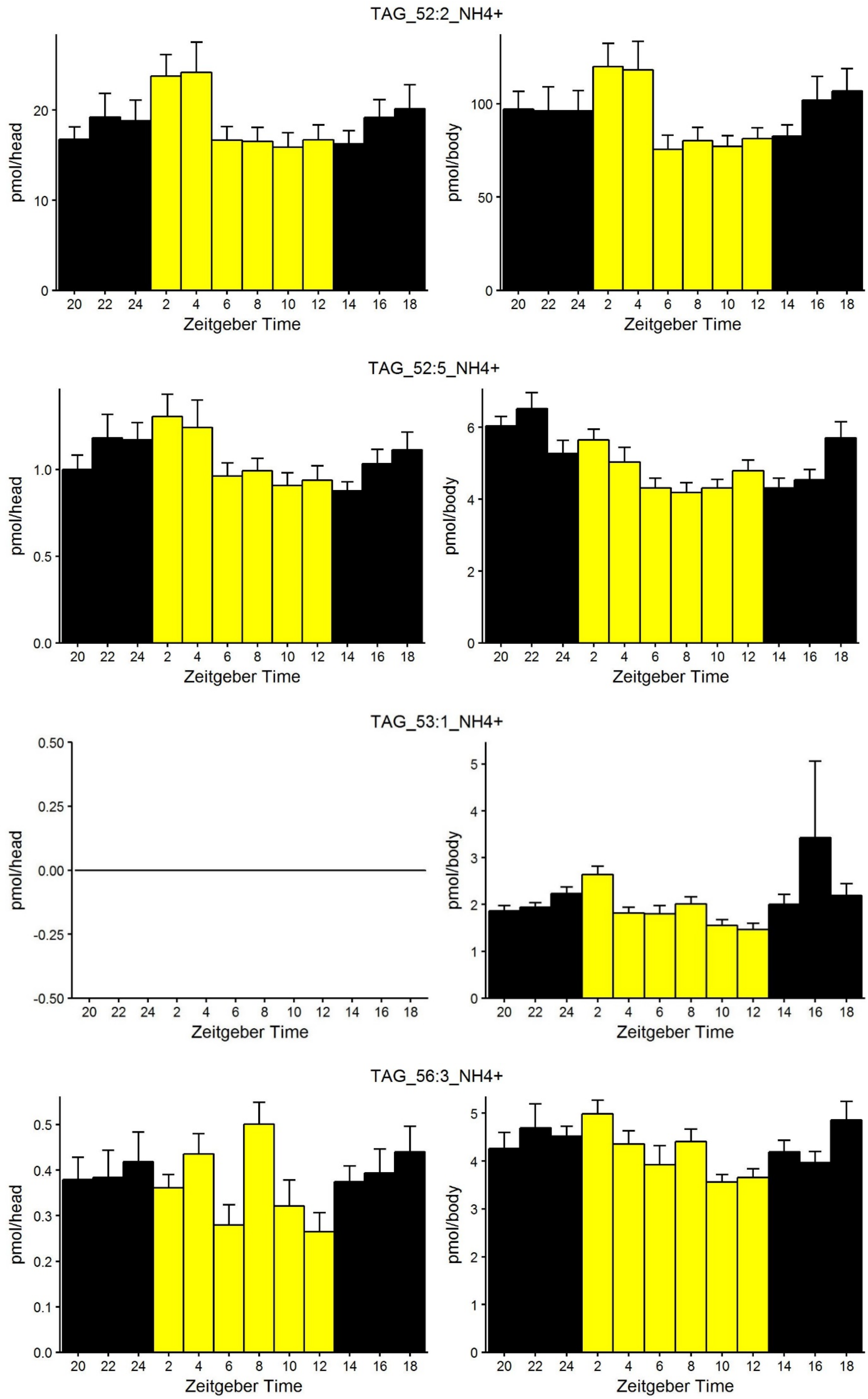

Suppl. Fig. 9 (continued) Daily variations of TAGs and DAGs in WT_{CS} under LD

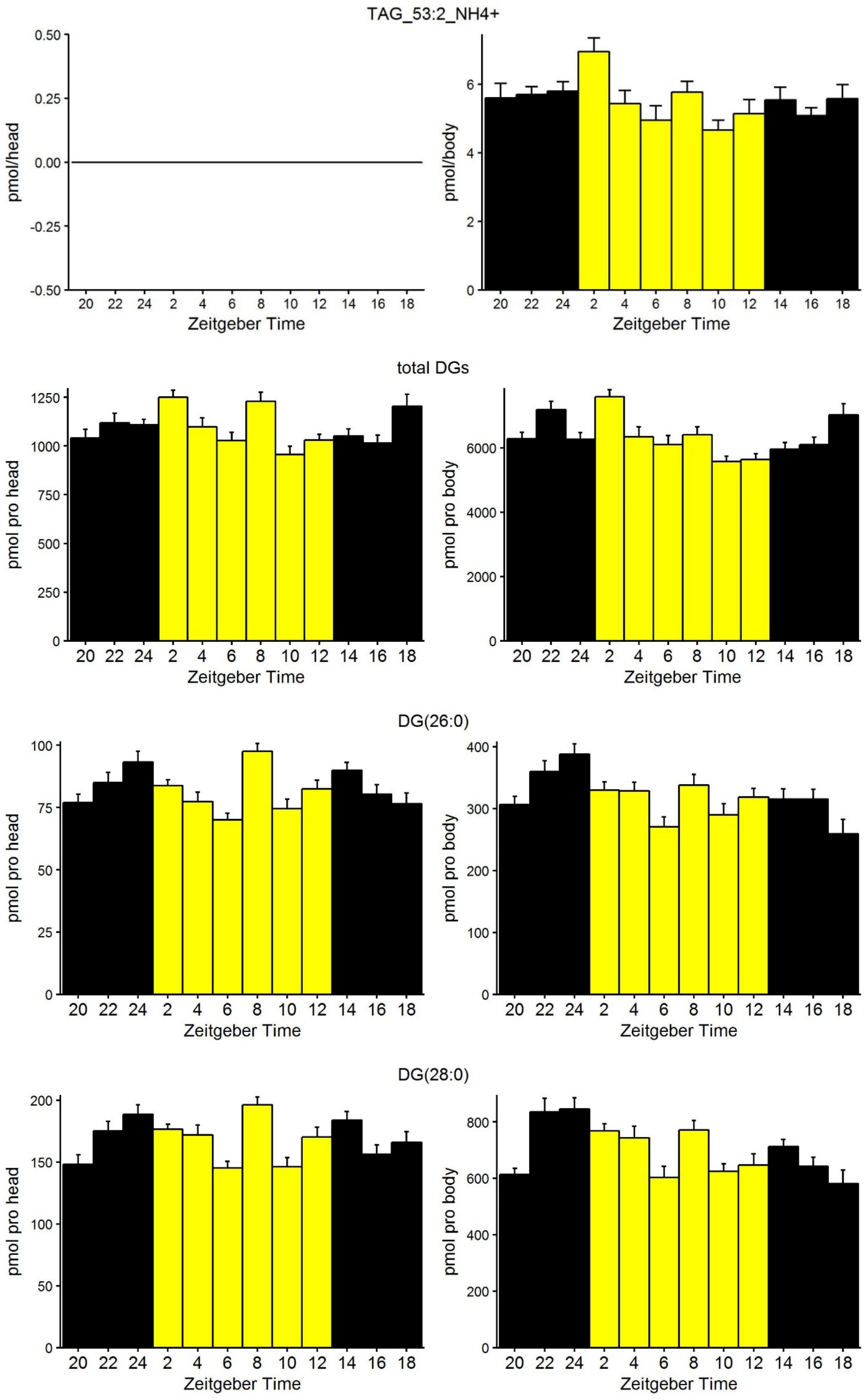

Suppl. Fig. 9 (continued) Daily variations of TAGs and DAGs in WT_{CS} under LD

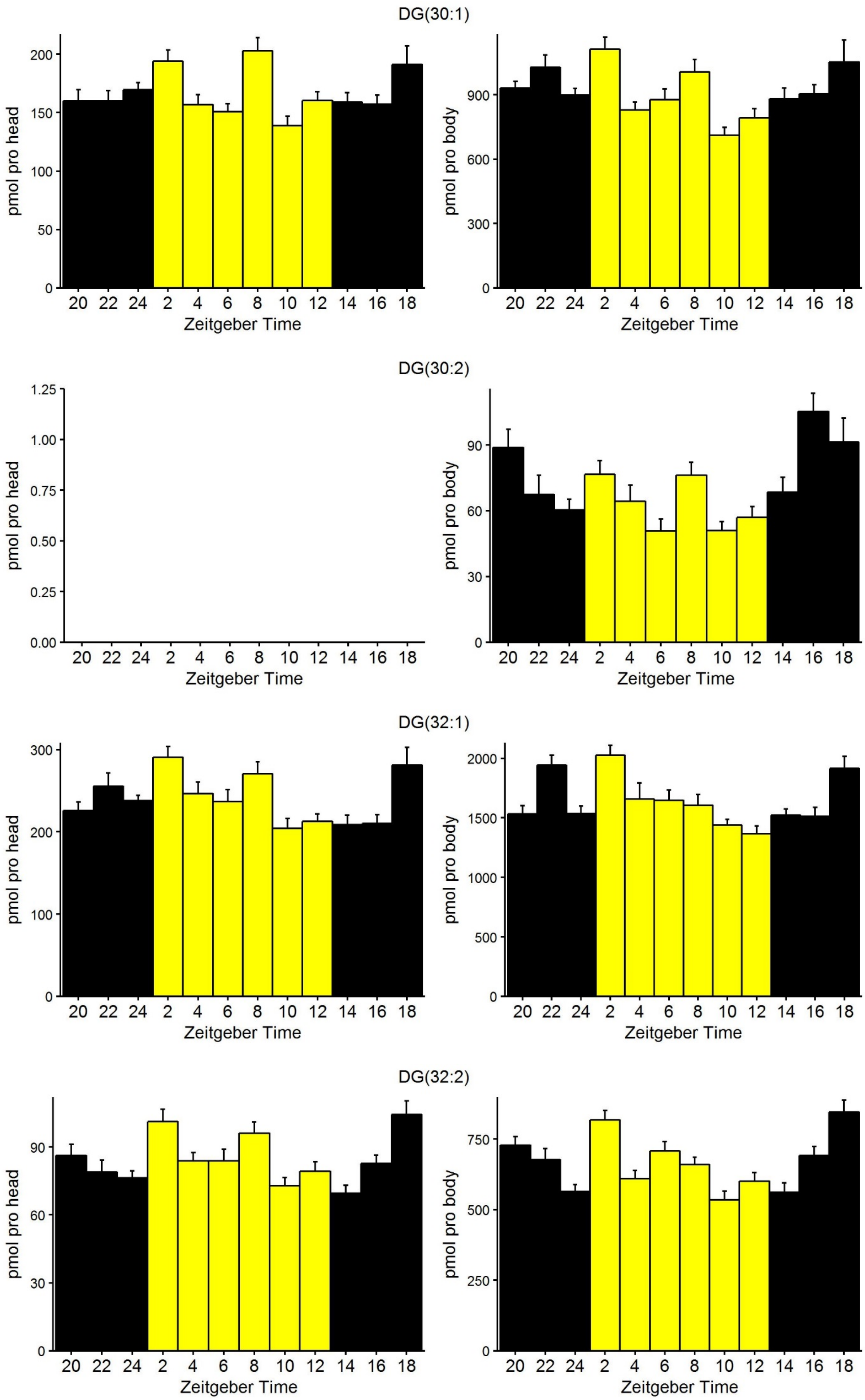

Suppl. Fig. 9 (continued) Daily variations of TAGs and DAGs in WT_{CS} under LD

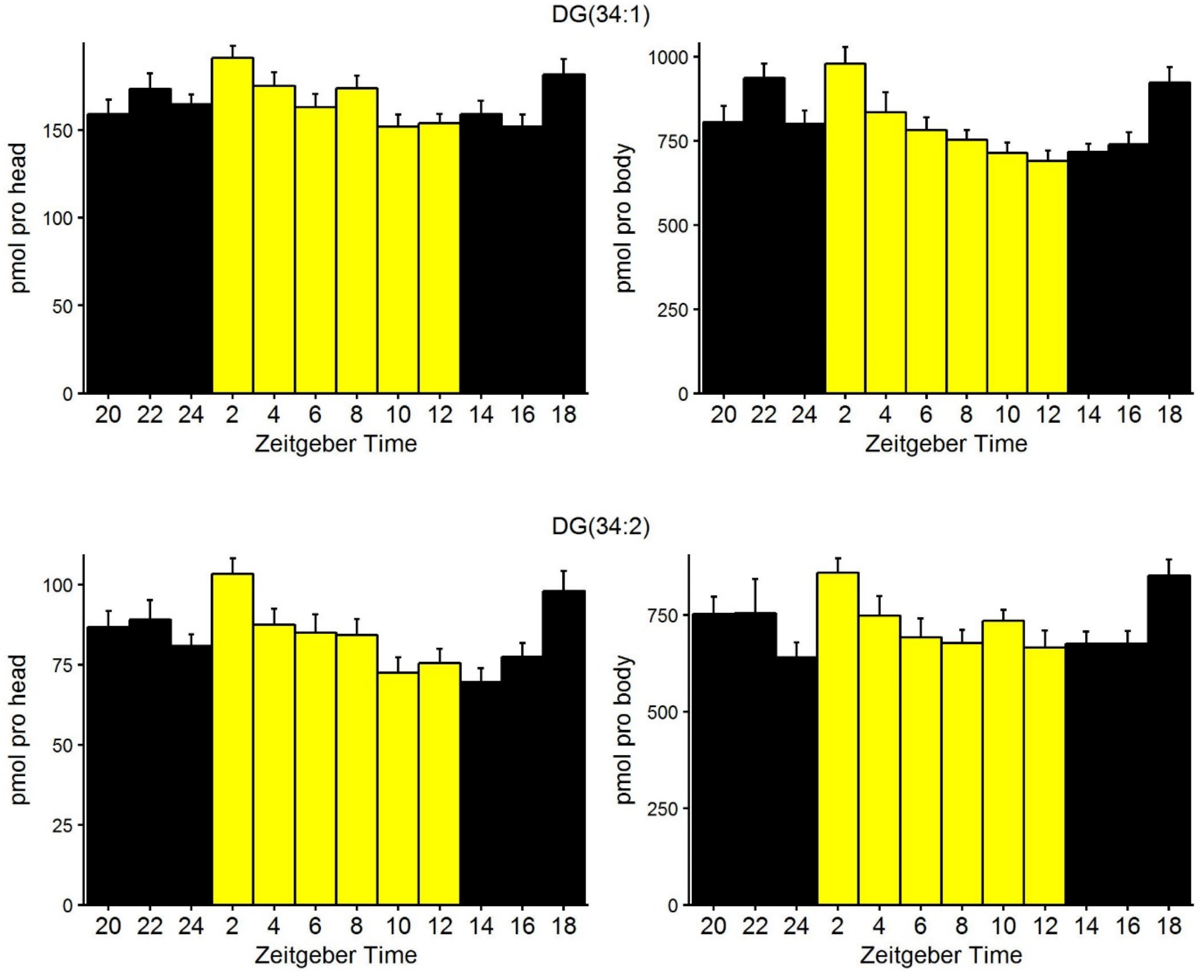

Supplementary Figure 9 Daily variations of TAGs and DAGs in WT_{CS} under LD. Levels of TAGs and DAGs, characterized by total number of carbons and total number of double bonds of the acyl chains, were determined as ammonium adduct in heads (left plot) and bodies (right plot) of wildtype flies. Samples were collected every two hours for three consecutive days and levels were averaged at each ZTs. Abbreviations: TAG_X:Y_NH4+ and DAG_X:Y_NH4+: ammonium adduct of triacylglycerols and diacylglycerols where X is the number of C atoms and Y is the number of double bonds in the fatty acids esterified to glycerol, line: non detected. Data represent means ± SE, n=4-7

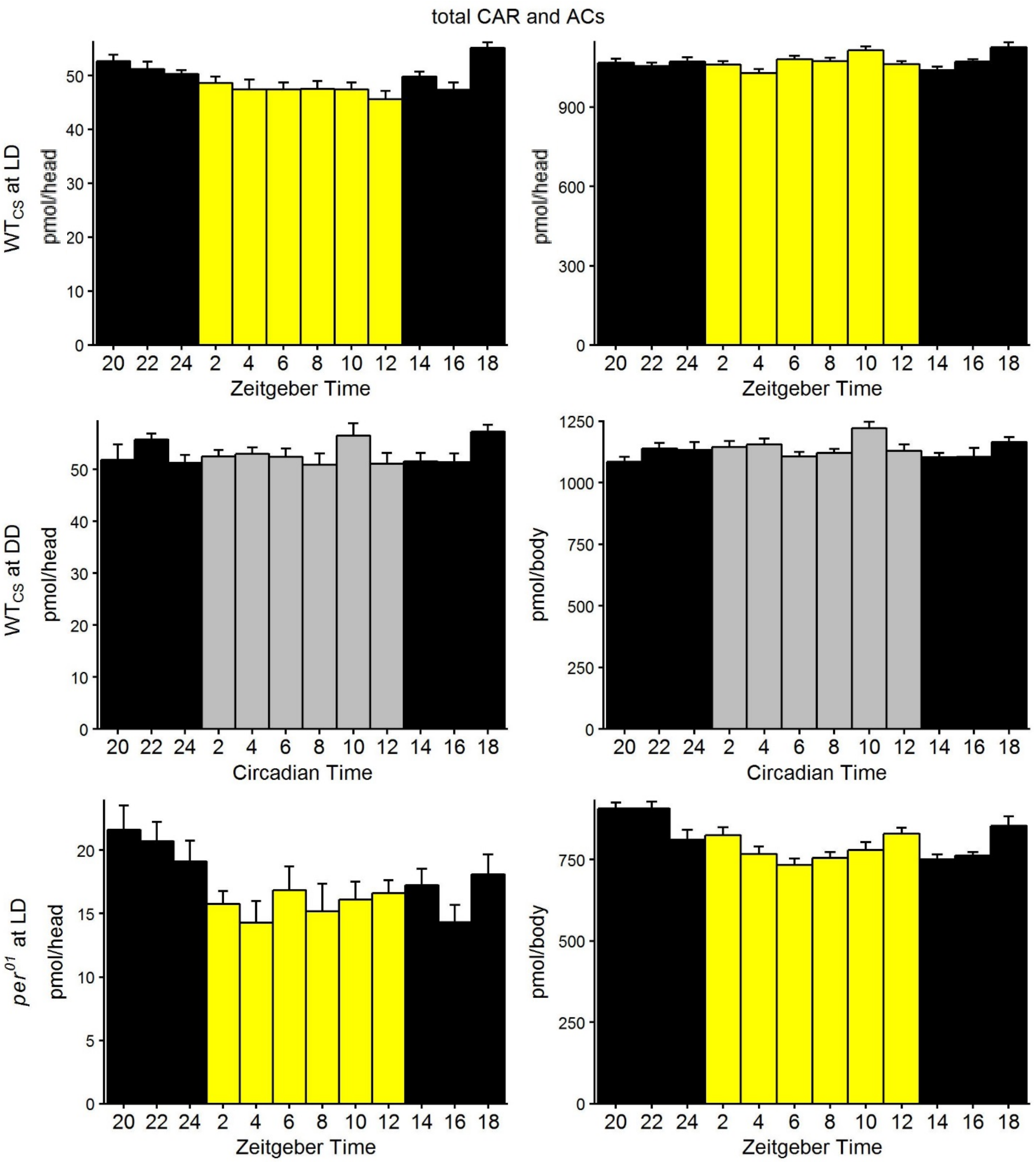
total CAR and ACs
WT_{CS} at LD
WT_{CS} at DD
per^{01} at LD
pmol/head
pmol/body
Zeitgeber Time
Circadian Time
20 22 24 2 4 6 8 10 12 14 16 18

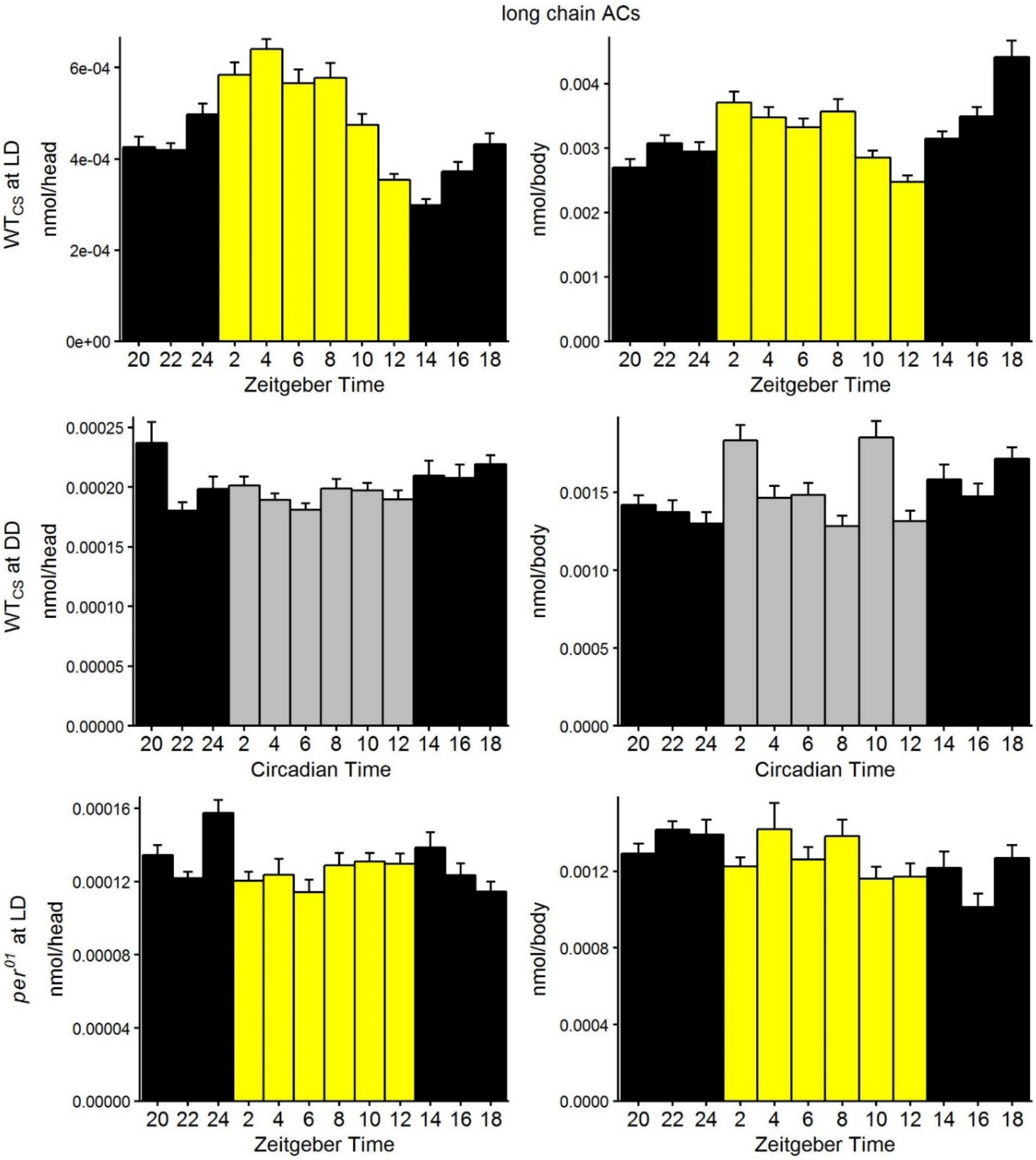

Suppl. Fig. 10 (continued) Daily variations of long chain ACs (fatty acyl carbon between 12-18) in WT_{CS} under LD and DD and in *per*^{01} under LD

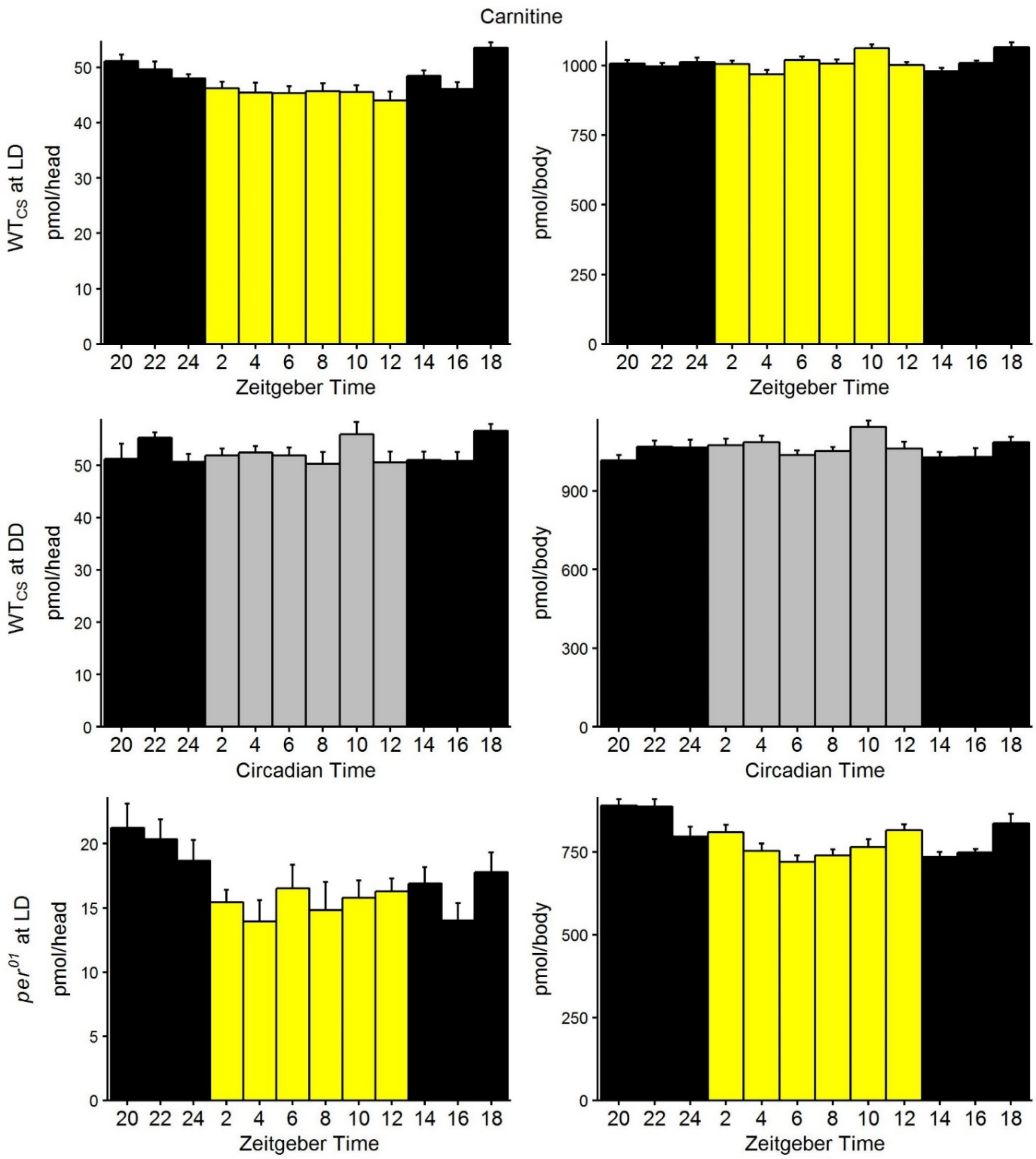

Suppl. Fig. 10 (continued) Daily variations of carnitine in WT_{CS} under LD and DD and in *per*^{01} under LD

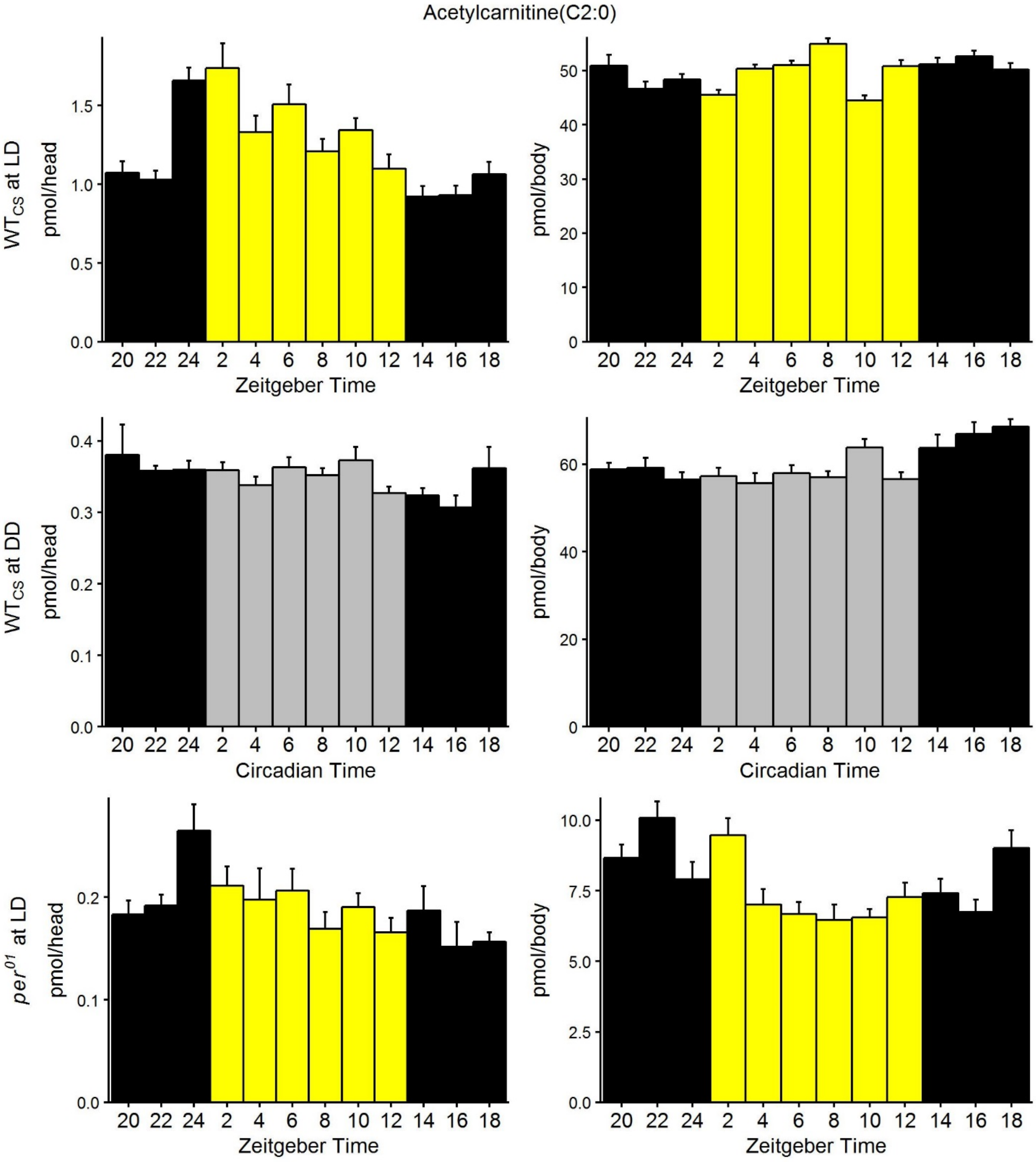

Suppl. Fig. 10 (continued): Daily variations of acetylcarnitine (AC(2:0)) in WT_{CS} under LD and DD and in *per*^{01} under LD

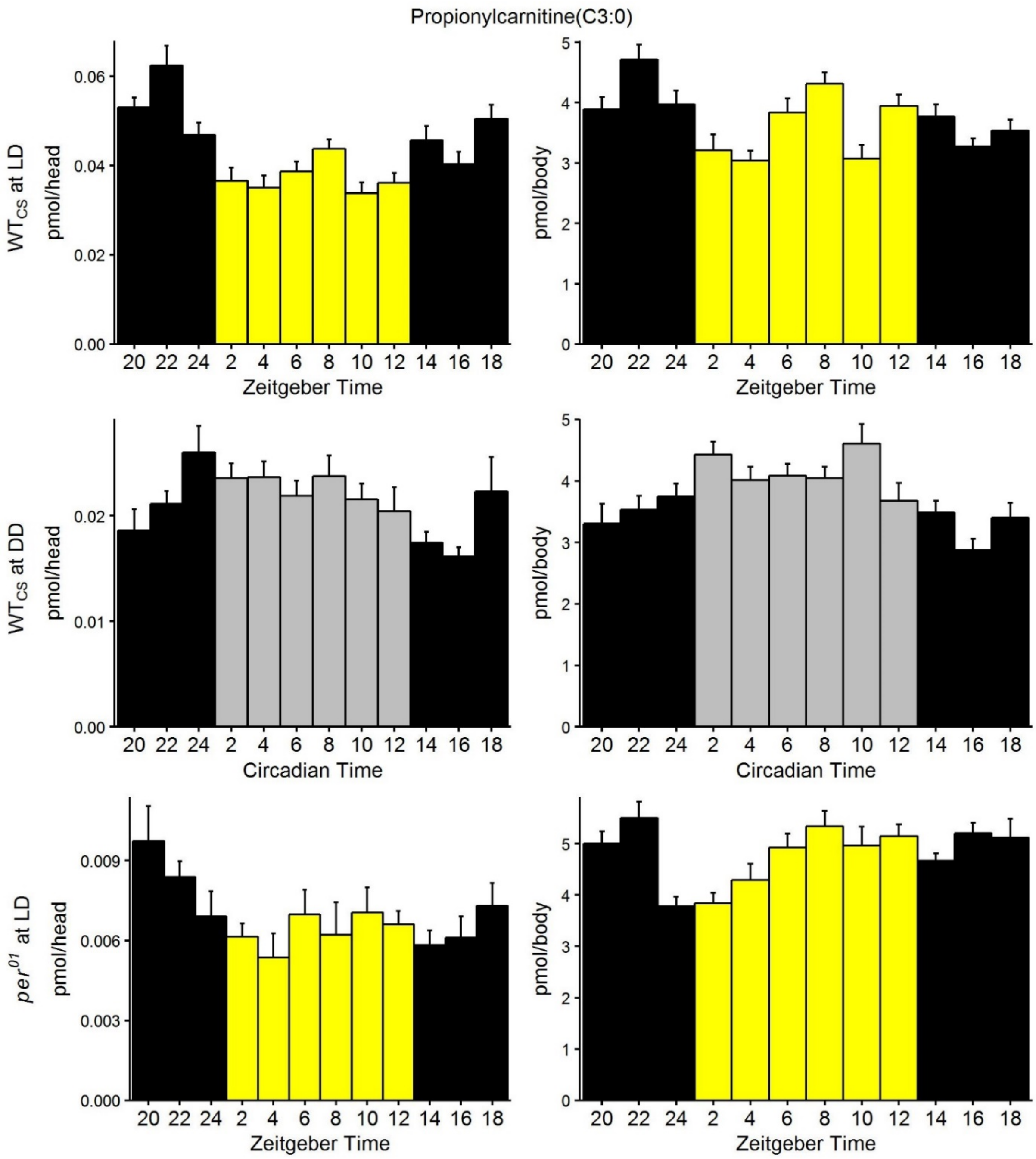

Suppl. Fig. 10 (continued): Daily variations of propinoylcarnitine (AC(3:0)) in WT$_{CS}$ under LD and DD and in *per*^{01} under LD

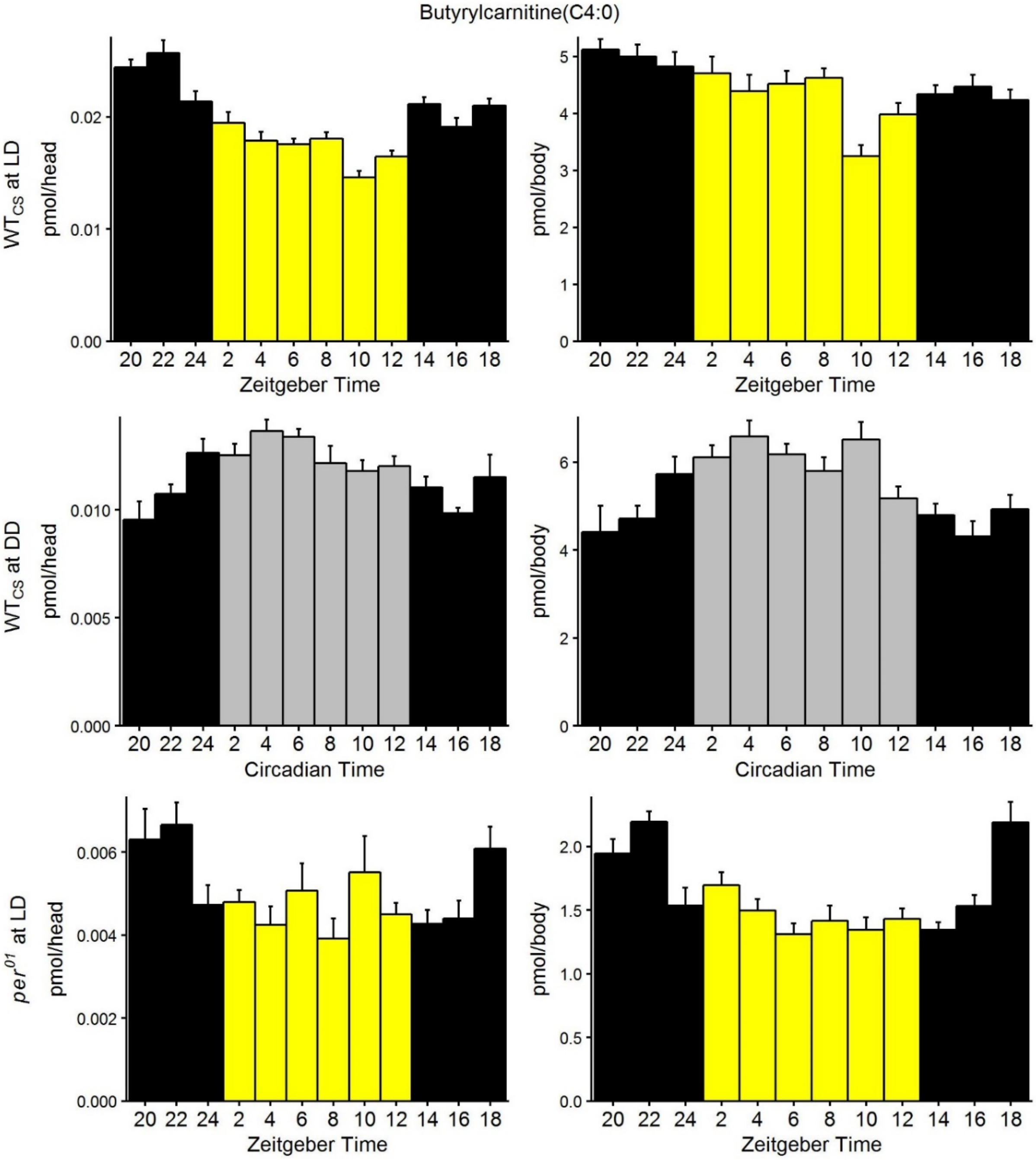

Suppl. Fig. 10 (continued): Daily variations of butanoylcarnitine (AC(4:0)) in WT_{CS} under LD and DD and in *per[01]* under LD

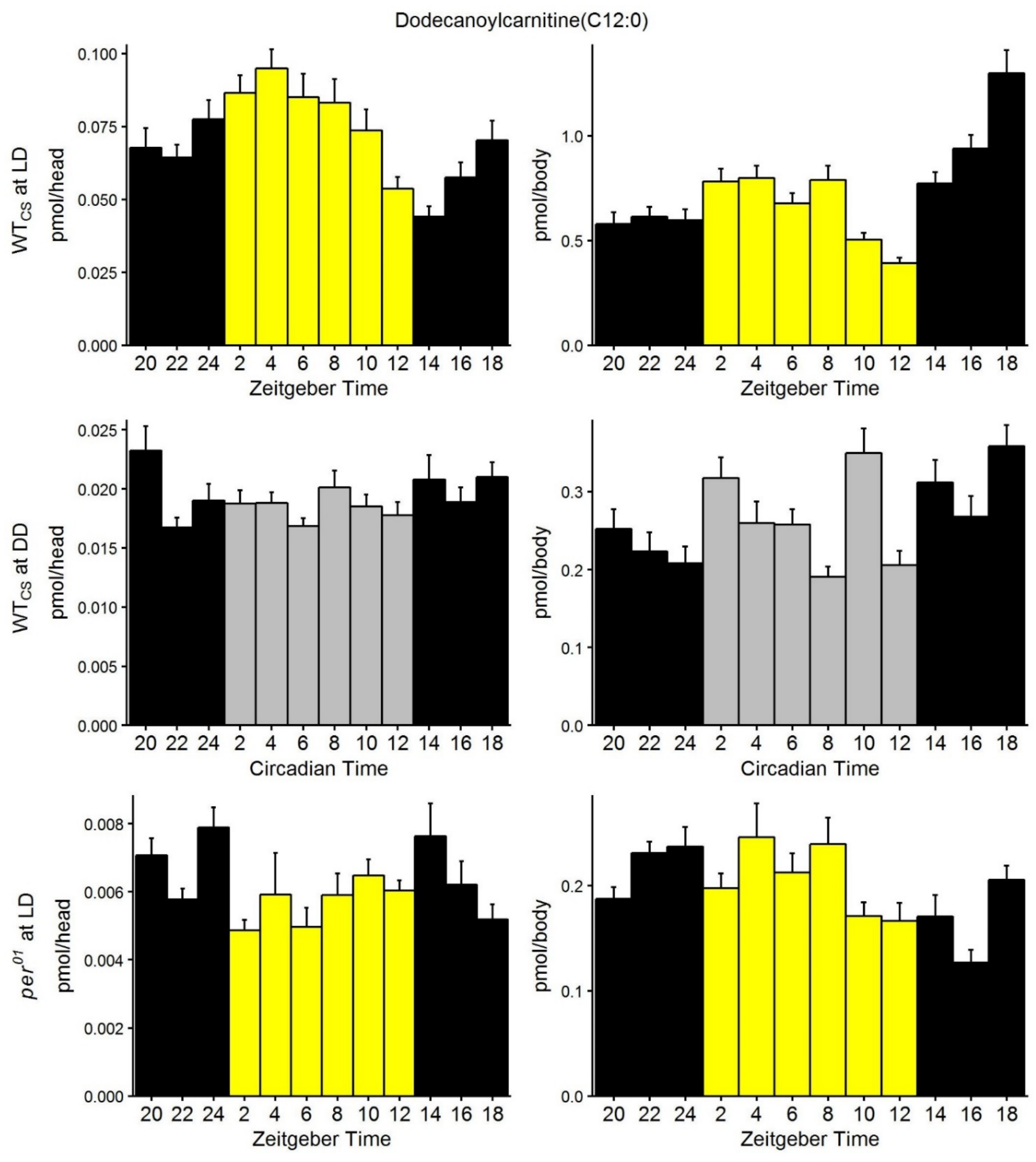

Suppl. Fig. 10 (continued): Daily variations of dodecanoyloylcarnitine (AC(12:0)) in WT_{CS} under LD and DD and in *per[01]* under LD

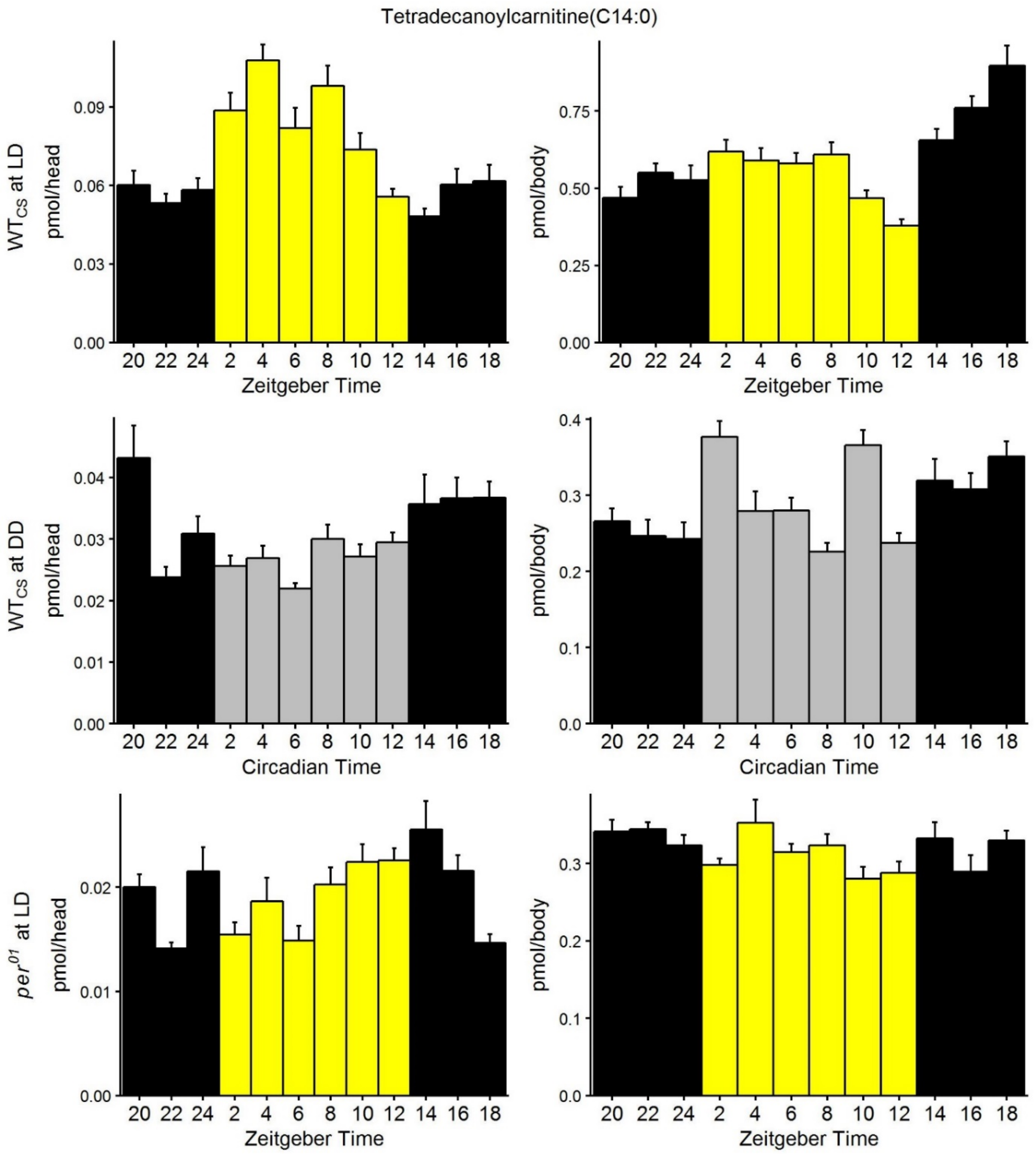

Suppl. Fig. 10 (continued): Daily variations of tetradecanoyloylcarnitine (AC(14:0)) in WT$_{CS}$ under LD and DD and in *per*^{01} under LD

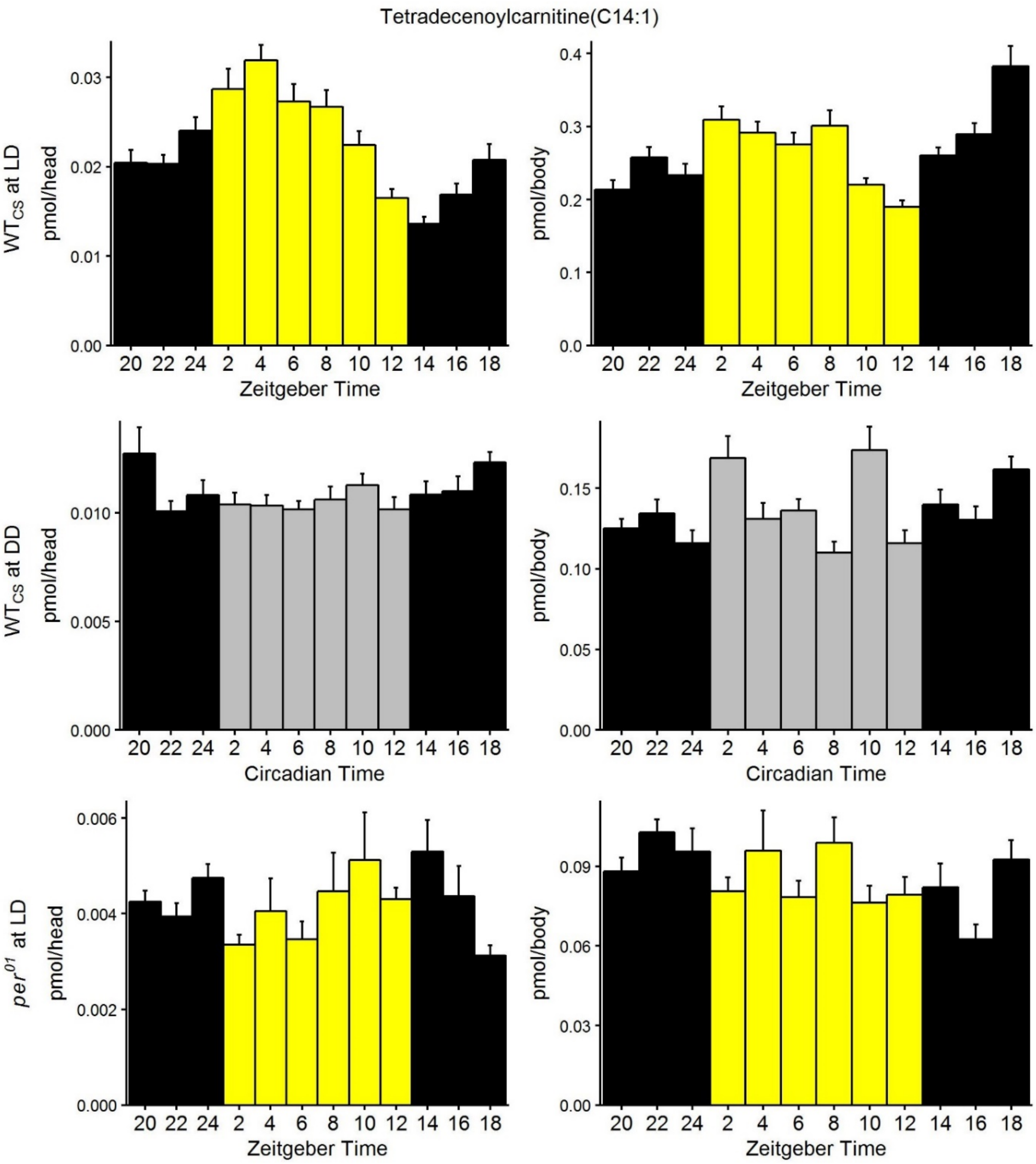

Suppl. Fig. 10 (continued): Daily variations of tetradecenoyloylcarnitine (AC(14:1)) in WT_{CS} under LD and DD and in *per*^{01} under LD

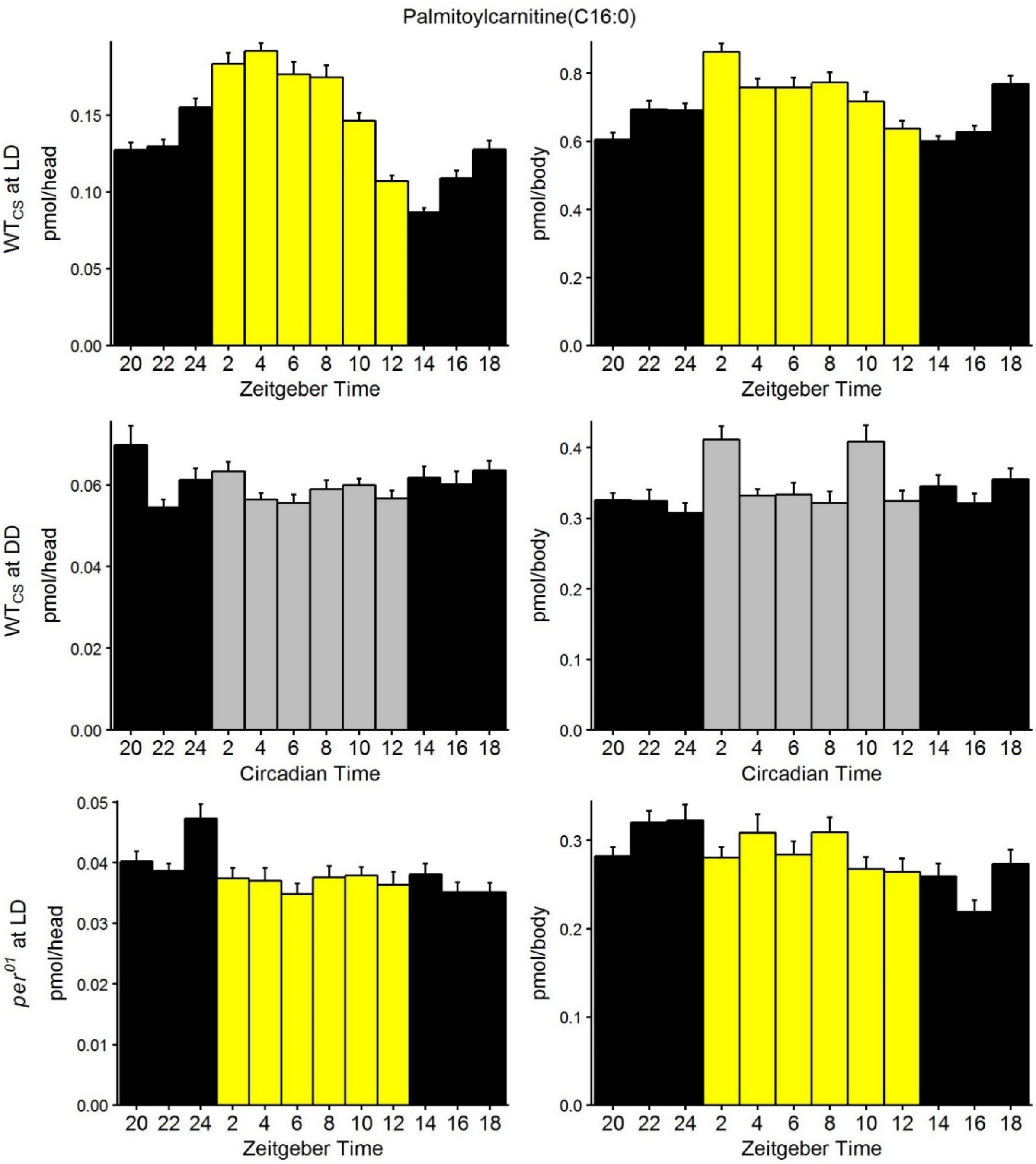

Suppl. Fig. 10 (continued): Daily variations of palmitoylcarnitine (AC(16:0)) in WT_{CS} under LD and DD and in *per*^{01} under LD

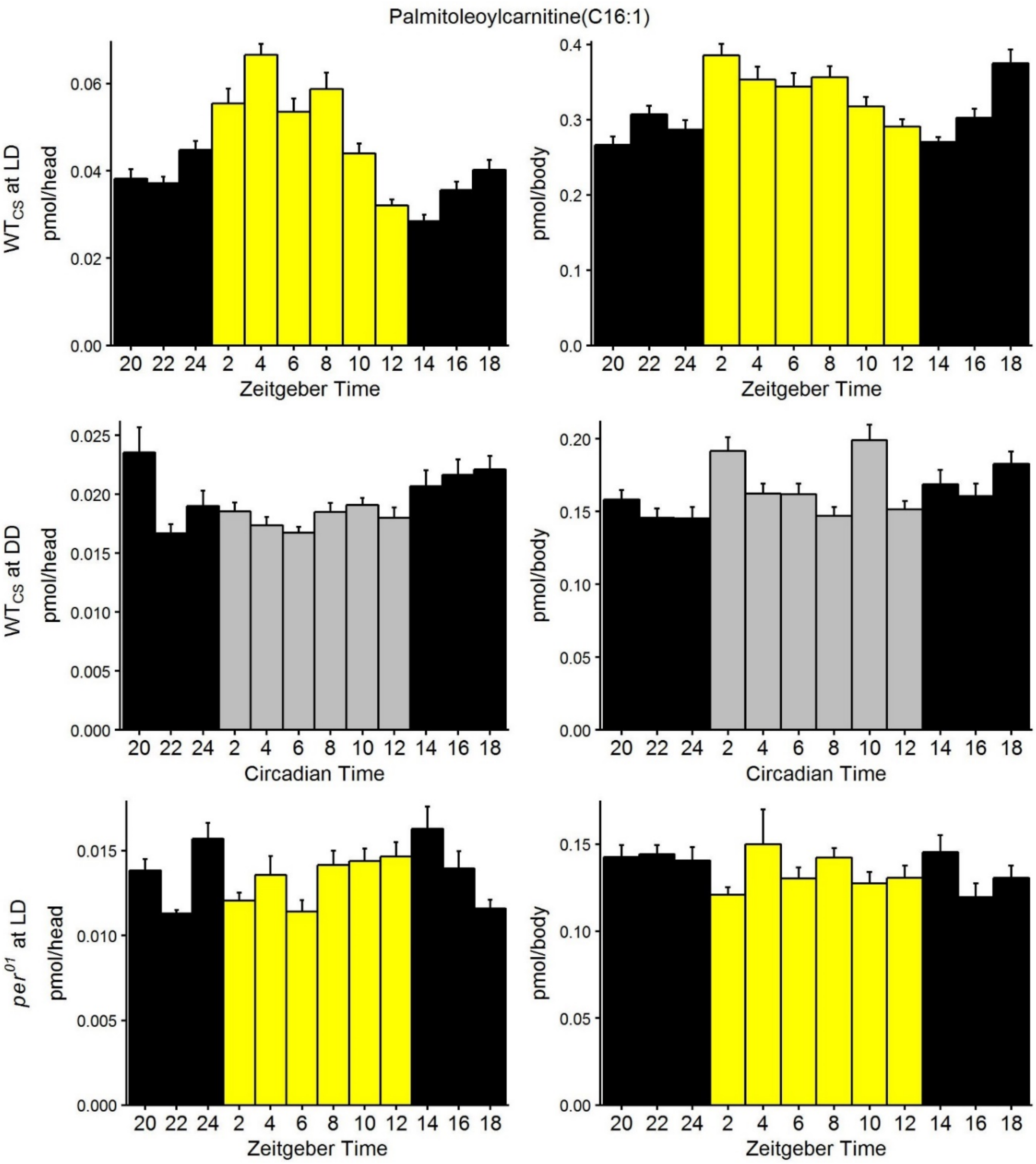

Suppl. Fig. 6 (continued): Daily variations of palmitleoylcarnitine (AC(16:1)) in WT_{CS} under LD and DD and in *per*^{01} under LD

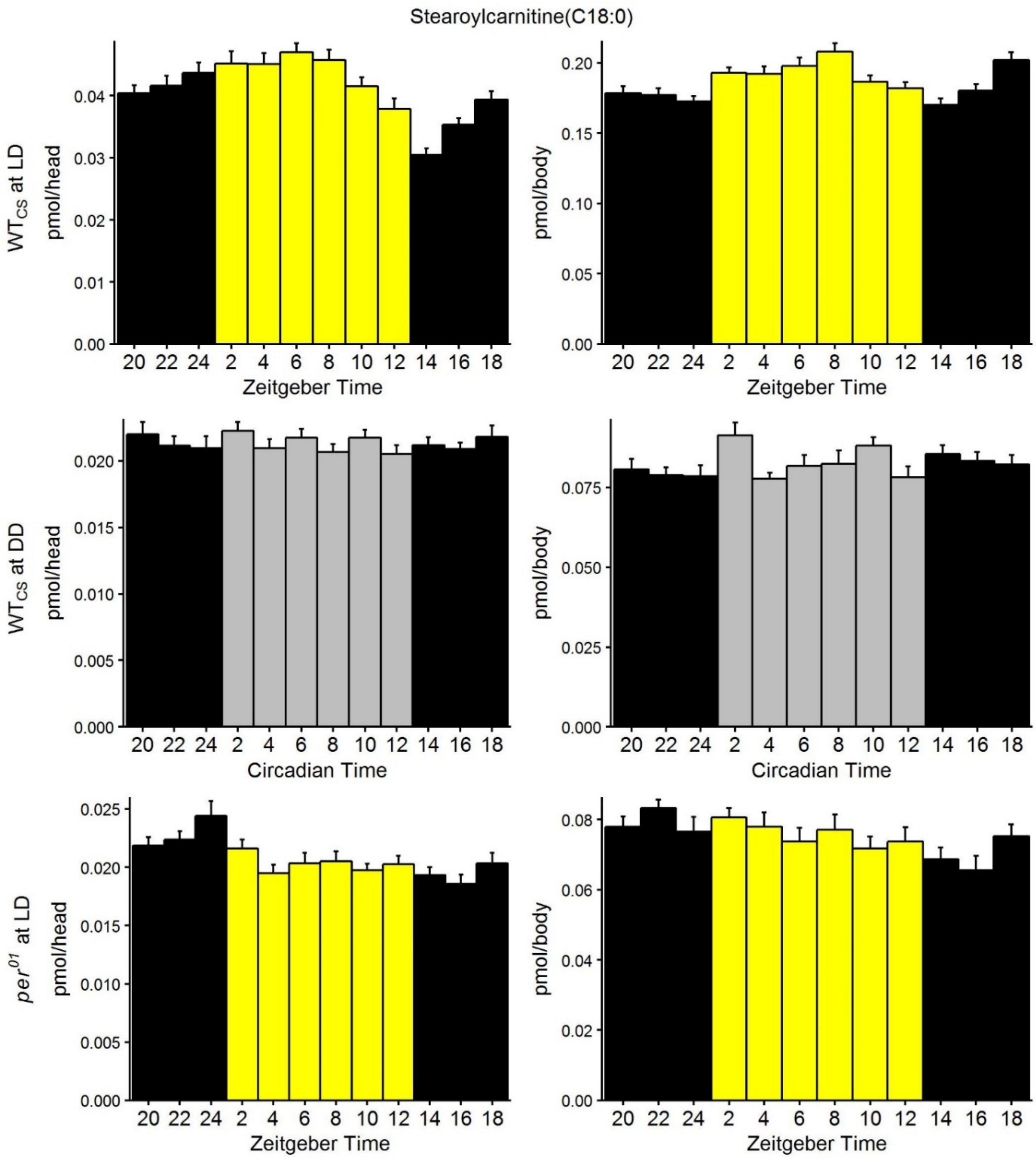

Suppl. Fig. 10 (continued): Daily variations of stearoylcarnitine (AC(18:0)) in WT_{CS} under LD and DD and in *per*^{01} under LD

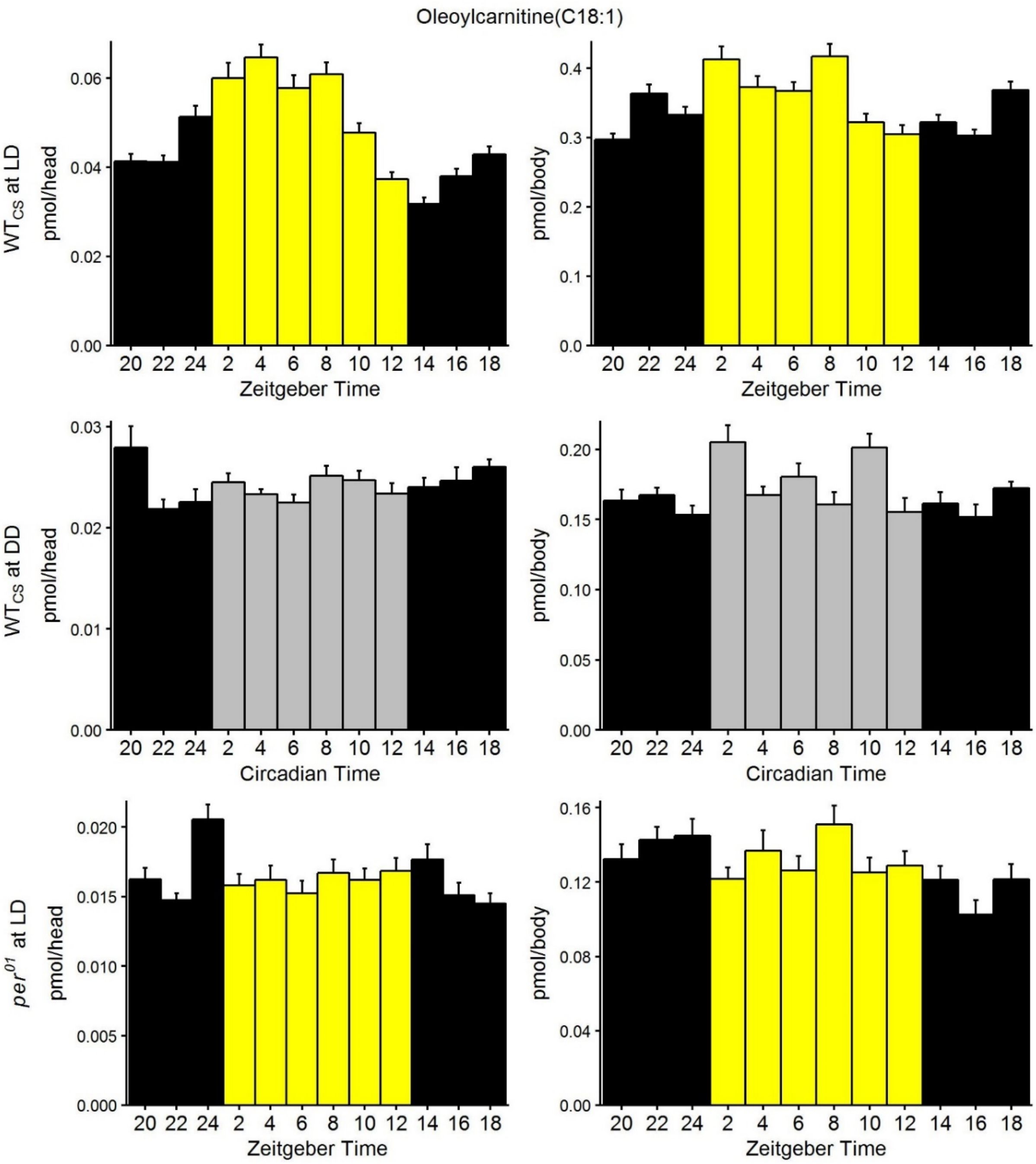

Suppl. Fig. 10 (continued): Daily variations of oleoylcarnitine (AC(18:1)) in WT_{CS} under LD and DD and in *per*^{01} under LD

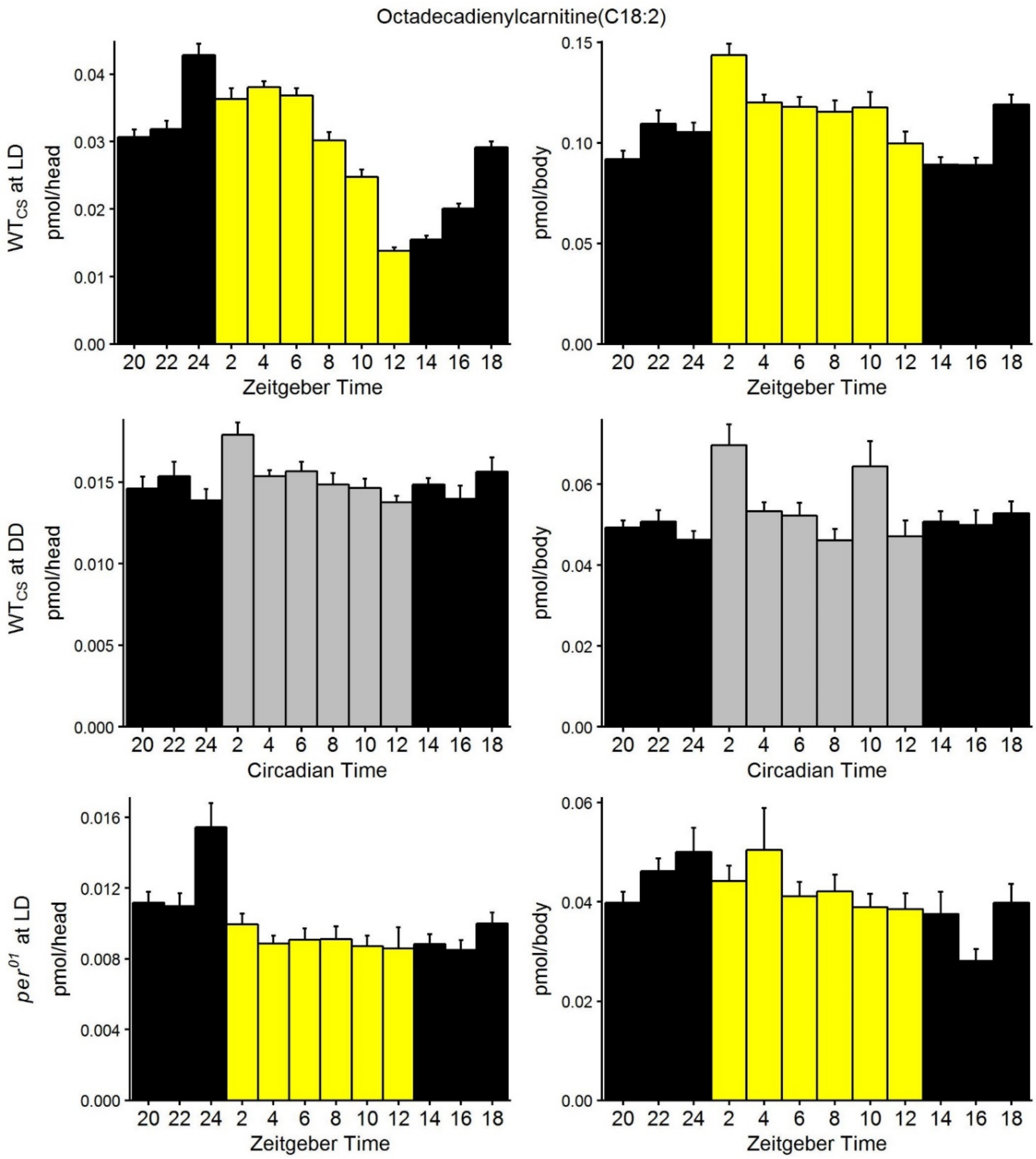

Suppl, Fig, 10: Daily variations of carnitine and ACs WT_{CS} under LD and DD and in *per*^{01} under LD. Levels of ACs, characterized by number of carbons and number of double bonds of fatty acid chains esterified to carnitine, were determined in heads (left plots) and bodies (right plots) of WT_{CS} under LD (top) and under DD (middle) and in *per*^{01} under LD (bottom). Samples were collected every two hours for three consecutive days at LD and for two days at DD and levels were averaged at each ZTs. Data represent means ± SE, n=4-7
